# Soil composition and rootstock genotype drive the root associated microbial communities in young grapevines

**DOI:** 10.3389/fmicb.2022.1031064

**Published:** 2022-11-10

**Authors:** Romain Darriaut, Livio Antonielli, Guilherme Martins, Patricia Ballestra, Philippe Vivin, Elisa Marguerit, Birgit Mitter, Isabelle Masneuf-Pomarède, Stéphane Compant, Nathalie Ollat, Virginie Lauvergeat

**Affiliations:** ^1^EGFV, Université de Bordeaux, Bordeaux Sciences Agro, Villenave d'Ornon, France; ^2^Bioresources Unit, Center for Health and Bioresources, AIT Austrian Institute of Technology GmbH, Tulln, Austria; ^3^Univ. Bordeaux, Bordeaux INP, INRAE, OENO, UMR 1366, ISVV, Villenave d’Ornon, France; ^4^Bordeaux Sciences Agro, Bordeaux INP, INRAE, OENO, UMR 1366, ISVV, Gradignan, France

**Keywords:** grapevine decline, microbiome, root endosphere, rhizosphere, arbuscular mycorrhizae

## Abstract

Soil microbiota plays a significant role in plant development and health and appears to be a major component of certain forms of grapevine decline. A greenhouse experiment was conducted to study the impact of the microbiological quality of the soil and grapevine rootstock genotype on the root microbial community and development of young plants. Two rootstocks heterografted with the same scion were grown in two vineyard soils differing in microbial composition and activities. After 4 months, culture-dependent approaches and amplicon sequencing of bacterial 16S rRNA gene and fungal ITS were performed on roots, rhizosphere and bulk soil samples. The root mycorrhizal colonization and number of cultivable microorganisms in the rhizosphere compartment of both genotypes were clearly influenced by the soil status. The fungal diversity and richness were dependent on the soil status and the rootstock, whereas bacterial richness was affected by the genotype only. Fungal genera associated with grapevine diseases were more abundant in declining soil and related root samples. The rootstock affected the compartmentalization of microbial communities, underscoring its influence on microorganism selection. Fluorescence *in situ* hybridization (FISH) confirmed the presence of predominant root-associated bacteria. These results emphasized the importance of rootstock genotype and soil composition in shaping the microbiome of young vines.

## Introduction

The microbiome, which is defined as the community of microorganisms and their theater of activity (Berg et al., 2020), is a biological indicator of plant health and productivity (Trivedi et al., 2020). The composition of the plant microbiota differs depending on the compartment studied, such as the phyllosphere, rhizosphere, or plant endosphere (Vandenkoornhuyse et al., 2015; Rossmann et al., 2017). The plant microbiota originates primarily from the surrounding soil, which is a reservoir of microorganisms that are attracted to the rhizosphere before they enter or do not enter the roots. The rhizosphere compartment, where most biogeochemical and nutrient cycling occurs, is considered a hotspot for microbial activity, whereas the plant endosphere contains microorganisms that interact intimately with the plant host (Reinhold-Hurek et al., 2015). Plant endophytes have received special attention since the functions of plant-associated microbiome can be either mutualistic or pathogenic to the host, which is determinant for crop health and productivity (Compant et al., 2021).

Abiotic parameters such as soil physicochemical characteristics (Hartman and Tringe, 2019), environmental conditions (Dubey et al., 2019), or even agricultural practices (Ke et al., 2021) alter the microorganism composition in the soil and therefore in the plant. Biotic factors such as pathogen invasion (Byers et al., 2020), and the application of plant growth-promoting rhizobacteria (Zhang et al., 2019) or mycorrhizal fungi (Zhou et al., 2020) can also modify telluric and plant endosphere microbial communities, in addition to plant age or genotype (Wagner et al., 2016).

Keystone microbial taxa, ecosystem engineers that greatly influence communities of microorganisms, are therefore essential to maintaining the health of the plant host (Banerjee et al., 2018). The depletion or the downsizing of these taxa in soil could cause microbial dysbiosis, which is responsible for the decline, and could be used to predict plant health (Wei et al., 2019). The term dysbiosis is primarily used in medical field, in which the diversity and stability of the microbiome serves as an indicator of certain pathologies. A recent study showed that the abundance of *Actinobacteria* and *Firmicutes* taxa in tomato plants was lower in the rhizosphere of diseased plants than in healthy ones (Lee et al., 2021).

Vineyard decline is described as an early decrease in vine productivity and sometimes as the premature, brutal or progressive death of the plant (Riou et al., 2016). This observation is often associated with individual or combined stresses, including biotic and abiotic factors. Most of the microbiome studies related to grapevine decline refer to grapevine trunk diseases (GTDs) that can be affiliated with diseases such as black-foot, Botryosphaeria dieback, Eutypa dieback, esca complex, and Petri disease (Gramaje et al., 2018; Cobos et al., 2022). Additionally, grapevine growth disturbance might be provoked by nutrient deficiencies or other abiotic stresses (Ollat et al., 2016). However, winegrowers are sometimes confronted with grapevine decline that is not associated with pathological causes or mineral imbalance but might be related to soil biological dysfunction (Darriaut et al., 2021, 2022).

Plant roots shape the rhizomicrobiome through their root exudates by selective enrichment of microbiota from the bulk soil, depending on the environmental conditions and their nutritional needs. The biochemical composition of the exudates varies according to the genotypes (species, varieties, or ecotypes), the developmental stage and the environment. Metabolite profiles of root exudates consequently drive the structure and function of the microbial communities of the plant (Vives-Peris et al., 2020; Chen et al., 2022; Koprivova and Kopriva, 2022; Seitz et al., 2022). In viticulture, *Vitis vinifera* varieties are mainly grown grafted onto the American *Vitis* species and hybrid rootstocks. These rootstock genotypes show different root and rhizosphere microbial communities sometimes influenced by the scion variety (Zarraonaindia et al., 2015; Marasco et al., 2018; Berlanas et al., 2019; Dries et al., 2021a; Vink et al., 2021; Marasco et al., 2022). Although studies have shown that in a few rootstocks, the exudate composition changes differently in response to nitrogen or iron deficiency (Cochetel et al., 2018; Marastoni et al., 2020), it has never been linked to the root microbiome diversity and functions of these genotypes.

Unproductive, dying, or dead vines are usually replaced by new young plants in the middle of producing rows in the vineyard. These young vines need at least 3 years to become productive. In the meantime, they select their root-associated microbiota through the rootstock. This period is crucial for the development and health of the future grapevine, especially in soil affected by unexplained decline. There is a lack of knowledge on the root microbiota selection process in young vines and the impact of the rootstock genotype. To our knowledge, no research has been carried out on the roots and soil microbiome of young, grafted vine plants grown with soil taken from vineyards in decline.

A survey of soil microbiome was previously conducted in a multisite study within vineyards affected by localized and unexplained decline (Darriaut et al., 2021). Despite relatively similar physicochemical features, lower enzymatic activities and different microbial profiles were found in soils supporting symptomatic vines (namely S soils) compared to soils presenting well-growing and asymptomatic vines (AS soils). The aim of the present work was to study how young, grafted plants from the nursery respond to the different microbiome compositions of soils from symptomatic (S) and asymptomatic (AS) areas of a vineyard affected by unexplained decline described in Darriaut et al. (2021). To evaluate the effect of the rootstock, grafted plants constituted on *Vitis vinifera* L. Cabernet Sauvignon grafted on either 1103P or RGM rootstocks were grown in pots filled with these soils in a 4-month greenhouse experiment. These two rootstocks are known to induce high and low scion vigor (1103P and RGM, respectively) and show a contrasted ability to tolerate drought, 1103P being more tolerant than RGM (Ibacache et al., 2020). They have been previously studied for their differential responses to nitrogen and phosphorus availability (Lecourt et al., 2015; Cochetel et al., 2017, 2018, 2019; Gautier et al., 2018). The microbial structural and functional diversity of bulk soil, rhizosphere and roots was assessed using a cultivable and molecular-based approach and revealed differences linked to the soil and rootstock.

## Materials and methods

### Plant material and greenhouse experiment

*V. vinife*ra L. cv. Cabernet Sauvignon (CS) scions were grafted on *Vitis riparia* cv. Riparia Gloire de Montpellier (RGM) or *Vitis berlandieri × Vitis rupestris* hybrid cv. 1103 Paulsen (1103P) rootstocks. One year-old grapevines were produced by the Pépinière Guillaume nursery (70,700, Charcenne, France) as traditional bare-root plants without any microbial addition. Before planting, 5 grams of roots of three plants per combination were sampled for 16S rRNA gene and ITS amplicon sequencing (Supplementary Figure 1).

Two soils were sampled in a vineyard from one Bordeaux region terroir (France), located in Graves’s appellation, described in Darriaut et al. (2021) as vineyard number 2. The physicochemical parameters of the soils, the different rootstock/scion combinations, and the vigour of the plants are described in Darriaut et al. (2021). Briefly, the vineyard has been planted in 2010, and is located on sandy soil under a sub-humid temperate climate with cool nights and a low risk of extreme temperatures, subtyped as Cfb in Köppen climate classification. An area displaying grapevine decline named symptomatic area (S), and an asymptomatic (healthy, called AS) area, distant from 20 meters from each other, were identified in the same plot. In the S areas, the high mortality (57% of dead or one-year old plants in S area compared to 1% in AS area) and low vigour of the living plants (2.2 times less pruning weight per vine in S area compared to AS area, *p* < 0.01) were not related to disease symptoms or the presence of the main viruses (GFLV or ArMV), and could not be explained by some soil mineral deficiencies (Darriaut et al., 2021). Soil samples from interrows (0–30 cm depth horizon) were collected in mid-April 2019 with a mini-excavator and sieved (mesh size <3 cm) to remove large roots and gravels. Pots (7.5 l) were filled with either S or AS soil and forty-five grafted plants from CS/1103P and CS/RGM combinations were planted per type of soil and put in the greenhouse. Then, CS/1103P and CS/RGM grafted plants were grown either on symptomatic (S-1103P and S-RGM samples, respectively) and asymptomatic vineyard soils (AS-1103P and AS-RGM samples, respectively). The 35 pots per condition were placed in a greenhouse and watered twice a week with 60 ml of tap water per pot. This experimental design was done twice, in 2019 and 2020, with new collection of soils from new proximal interrow, and new nursery plant material (Supplementary Figure 1).

### Plant phenotyping and sampling of roots and soils

After 4.5 months of growth, chlorophyll contents of the top fourth and third leaves were measured using a portable chlorophyll meter (SPAD-502, Konica Minolta Sensing, Inc., Japan) on ten biological replicates per condition. Measurements of stem and trunk length and diameter were recorded on the same plants. Thereafter, the dry weight of total leaves, stems, trunk and roots was evaluated after drying at 70°C for 72 h (*n* = 10 individuals).

Roots, rhizosphere, and bulk soil were collected from each pot of three biological replicates (Supplementary Figure 1). Soil aggregates were removed from the roots by manual shaking. Approximately 5 grams of roots were sampled in tubes containing sterile 0.85% NaCl solution and vortexed prior to 5,000 g centrifugation for 10 min to detach the rhizosphere from the roots. Half of each root sample was surface sterilized with 3% hypochlorite sodium for 1 min subsequently to 3% H_2_O_2_ for 1 min and rinsed thrice with sterile water. These surface sterilized roots were stored at −80°C prior to DNA extraction and FISH visualization (*n* = 3). The other half of roots was used at fresh state for staining of mycorrhizal structures.

Rhizosphere samples were weighted and divided into two parts. The first one was lyophilized for 48 h using Christ Alpha® 1–4 (Bioblock Scientific) and stored at −80°C prior to DNA extraction. The second part was used for the potential metabolic diversity (PMD), the isolates quantification with plating method, as well as the isolates identification through MALDI-TOF-MS. Bulk soils from the pots and three samples of each vineyard soils (sampled as described in Darriaut et al., 2021) were lyophilized and stored at −80°C.

### DNA extraction

DNA was isolated using FastDNA Spin kit for soil (MP Biomedicals) following manufacturer’s instructions except that bead beating step on FastPrep device and aspiration of liquid samples were performed twice. Bead beating power on FastPrep device was set on power 5 for 30 s for soils and to power 6 for 40 s for root samples. DNA was isolated from 500 mg of lyophilized soils and 200 mg of roots previously grounded in liquid nitrogen and pulverized by bead beating in steel containers on a Retsch mill. DNAs were eluted from DNA binding matrix with 100 μl of sterile H_2_O. For q-PCR measurements, DNA was isolated from 250 mg of rhizosphere soil as previously described in Darriaut et al. (2021), using DNeasy PowerSoil Pro kit (Qiagen).

### Potential metabolic diversity coupled to quantification of microorganisms, and mycorrhizal root colonization

PMD, quantification of cultivable bacteria and fungi, and quantitative PCR (qPCR) of bacterial 16S, archaeal 16S, and fungal 18S rRNA genes were done according to Darriaut et al. (2021). Briefly, R2A (Reasoner’s 2A agar) medium amended with 25 mg/l of nystatin and PDA (Potato Dextrose Agar) supplemented with 500 mg/l of gentamicin and 50 mg/l of chloramphenicol were used to quantify cultivable bacteria and fungi, respectively, in five replicates per rhizosphere samples. In parallel, the Biolog Eco-Plates™ system was used to assess the PMD by inoculating three rhizosphere samples per condition in walls of these plates, which were 1:1000 water-diluted. Eco-Plates were incubated at 20°C in the dark and their absorbance was measured at 590 nm every 24 h for 5 days. Global microbial metabolic activity in each replicate was expressed as the Average Well Color Development (AWCD), which permit to calculate the functional microbial richness (number of utilized substrates as the higher AWCD mean among the tested soils at 96 h), Simpson’s index, and the area under AWCD curve (AUC). Regarding the qPCR, they were based on absolute quantification of genes amplified from a PCR using the universal 341F/515R bacterial, Arch967F/Arch1060R archaeal, and FF390/FR1 fungal primers. The genes were subcloned using the pGEM®-T easy vector system (Promega) and the qPCR were performed in three replicates per samples using the GoTaq® qPCR Master Mix (Promega).

The presence of arbuscular mycorrhizal structures was estimated in roots of the second-year experiment (2020). Fresh roots were stained by the ink-KOH-H_2_O_2_ method modified from Phillips and Hayman (1970). Briefly, fresh roots were rinsed in sterile water and incubated in 10% KOH for 30 min at 95°C. Immediately after the incubation, 3% H_2_O_2_ has been added to the mixture. After few minutes, the roots were rinsed thrice with sterile water and stained by incubating at 90°C for 5 min in 5% India ink (Super Black™) / 8% acetic acid solution. Roots were destained at ambient temperature with 8% acetic acid for 15 min before washed with sterile water. Thirty samples of 1 cm of stained roots were placed on glass slices with glycerol and observed with a light microscope LEICA DM750 equipped with a LEICA ICC50 W camera. Arbuscular mycorrhizal colonization was estimated with Trouvelot et al. (1986) method and Mycocalc program.^1^

### Identification of bacterial isolates through MALDI-TOF-MS

Eight hundred bacterial isolates in total were randomly selected among colonies forming units (CFUs) grown on R2A plates previously inoculated with rhizosphere samples (100 CFUs × 4 conditions × 2 years). Each fresh isolate was smeared on MSP96 target polished steel BC plate and overlaid with 1 μl of 70% formic acid. Once dried at room temperature, samples were overlaid with MALDI matrix (1 μl, 10 mg/ml of α-cyano-4-hydroxycinnamic acid in 50% acetonitrile/2.5% trifluoroacetic acid) for crystallization (Windholtz et al., 2021). Once dried, the target plate was submitted to MALDI-TOF-MS analysis using Microflex MALDI-TOF (Bruker Daltonik GmbH, Leipzig, Germany) bench-top mass spectrometer scanned with laser wavelength of 337 nm and acceleration voltage of 20 kV. The analysis was performed using Flex Control, MTB Compass, and MALDI-Biotyper™ software (Bruker Daltonics, Germany) by comparing the mass profile of the isolates to mass profiles in the Biotyper database. Bacterial test standard was added to every plate in order to calibrate the mass spectral data performed by the MALDI-TOF-MS. Mass profiles matching were obtained as score values and ranged from 0 to 3 as indicated by the manufacturer. Score values above 2.2 corresponded to highly probable species identification, the ones between 1.8 and 2 displayed identifications at the genus level, while score values below 1.8 were not considered as trustful identifications.

### Amplicon libraries preparation and sequencing

All PCR amplifications were carried out by KAPA HiFi HotStart PCR Kit (Roche) mixture containing template DNA, 1x KAPA HiFi buffer with magnesium, 300 μM dNTPs, 0.25 units of KAPA HiFi polymerase, and specified concentration of primers. Primers are listed in Supplementary Table 1 and cycling conditions in Supplementary Table 2. PCR amplifications on 1:10 diluted DNA of each sample was repeated three times and amplicons pooled together for further indexing.

Amplification of 16S rRNA gene was done with 300 nM of primers 799f/1175r, designed to amplify V5-V7 bacterial regions with the exclusion of chloroplast DNA (Chelius and Triplett, 2001). PCR bands were excised and separated from plant mitochondrial amplicons for further indexing.

Two internal transcribed spacer region (ITS) libraries were created to sequence ITS1 and ITS2 based on primers listed in Supplementary Table 1. PCR amplification for the first ITS library which target ITS1 region was performed with 500 nM of primer 5.8S-Fun_NeXTf coupled to reverse primers [ITS5_Mix = ITS4-Fun_NeXTr + ITS43S-Fun_NeXTr, adapted from Taylor et al. (2016)]. Second ITS library, targeting ITS2, was created by applying nested PCR approach. First PCR amplification was performed with 300 nM of primers ITS1F/TW13 (Klaubauf et al., 2010), designed to amplify fungal ITS and part of fungal large subunit (LSU). This was followed by the second amplification using 450 nM of primers mixes containing forward (ITS3_Mix = ITS31_NeXTf + ITS32_NeXTf + ITS33_NeXTf + ITS34_NeXTf + ITS35_NeXTf) and reverse primers (ITS4_Mix = ITS4_NeXTr + ITS43S_NeXTr) on 3 μl of the first PCR amplicon (Tedersoo et al., 2014).

Indexing-PCR of 16S rDNA and ITS DNA amplicons was performed using Illumina Nextera XT indexing primers (forward S502-S503, S505-S508, S510-S511 and reverse N701-N707, N710-N712, N714-N715) under following conditions: 1 μl of 16S rRNA gene or ITS PCR amplicons (each derived from three pooled independent PCR amplifications), 1x KAPA HiFi buffer with 2 mM MgCl_2_, 300 μM dNTPs, 300 nM of each forward and reverse indexing primer, 0.25 unit of KAPA HiFi polymerase and H_2_O up to 50 μl. Amplification was performed making initial denaturation step at 95°C for 3 min, 12 cycles including denaturation at 95°C for 30 s, annealing at 60°C for 30 s and elongation at 72°C for 30 s, and final elongation at 72°C for 5 min.

Intensity of bands was measured and compared using Image Lab 6.1 software (BioRad). Amplicons were then mixed in equimolar amounts to create pooled libraries. Libraries were cleaned first by extraction with Phenol-Chloroform-Isoamyl alcohol (24:24:1) and Chloroform-Isoamyl alcohol (24:1) followed by the spin filtration using Amicon Ultracel 30 K centrifugal filters (Millipore UFC503096) applying 2 × 500 μl ddH_2_O and finally using AmPure XP magnetic beads (Beckman Coulter) according to the manufacturer instruction. Two libraries, based on either soils or root samples were created, and sequenced separately. For sequencing 6 pM library was spiked with 8% PhiX and sequencing was performed on the MiSeq System (Illumina) using Illumina MiSeq® Reagent Kit v3 (600 cycle) (MS-102-3,003).

### 16S rRNA gene and ITS sequencing pre-processing

MiSeq sequences were filtered with Bowtie 2 v.2.3.4.3 (Langmead and Salzberg, 2012) to remove PhiX control reads, if still present, and sequence quality was preliminarily checked with FastQC v.0.11.8 (Andrews, 2010). Primers were removed using Cutadapt v.1.18 (Martin, 2011). Sequences were quality filtered, trimmed, denoised and amplicon sequence variants (ASVs) were generated with DADA2 v1.20.0 (Callahan et al., 2016). Denoised forward and reverse ASV sequences were merged, and chimeras were removed. Filtered ASVs were checked using Metaxa2 v2.2.3 (Bengtsson-Palme et al., 2016) and ITSx v1.1.3 (Bengtsson-Palme et al., 2013) for targeting the presence of V5-V7 16S rRNA and ITS2 region, in archaeal and bacterial sequences and fungal sequences, respectively. Taxonomic assignment of 16S rRNA gene ASVs and ITS based ASVs was performed using the RDP classifier of DADA2 against the SILVA v138 database (Quast et al., 2012) and UNITE 8.2 database (Nilsson et al., 2019), respectively. After taxonomic classification, ASVs classified as other than archaea, bacteria or fungi were removed.

### Bioinformatic analysis and statistics

All analysis and graphs were performed on R (R-4.1.2) using RStudio (2021.9.1.372). Figures were generated with *ggplot2* (3.3.5) and *ggthemes* (4.2.4) packages and arranged using *ggpubr* (version 0.4.0) (Wickham and Chang, 2008; Kassambara, 2020; Arnold, 2021). Two-way Analysis of Variance (ANOVA) with soil status (AS or S) and rootstock genotype (RGM or 1103P) factors were performed on cultivable, q-PCR and Eco-Plates measurements. Residuals were checked for their independency, normality, and variance homogeneity with the Durbin Watson, Shapiro–Wilk, and Bartlett tests, respectively. When assumptions for parametric tests were not respected, a multiple pairwise comparison using Wilcoxon test was performed subsequently to Kruskal–Wallis test using the *multcomp* (1.4–18) package (Hothorn et al., 2008). Principal Component Analysis was performed using *FactoMineR* (2.4) and *missMDA* (1.18) while Venn diagrams were generated using *VennDiagram* (1.7.1) (Le et al., 2008; Josse and Husson, 2016; Chen, 2021).

Regarding the amplicon-based sequencing data, low abundant ASVs with a maximum relative abundance below 0.1% per sample were discarded using “filter.OTU” function from *RAM* package (1.2.1.7) (Chen et al., 2018). Richness (observed ASVs) and diversity (Simpson’s index) values were calculated employing the *rtk* (0.2.6.1) package, averaging the results obtained after 999 rarefactions (Saary et al., 2017). Richness and diversity metrics were compared between compartments, rootstock genotype, year of experiment, and soil status by means of pairwise comparisons from *RVAideMemoire* (0.9–81) (Hervé, 2021). Prior to any beta-diversity calculation, differences in sequencing depth were addressed applying the median of ratios method implemented in the DESeq2 Bioconductor package (Love et al., 2021). The differences between microbial communities were investigated using Bray–Curtis dissimilarity distance. Multivariate analysis of bacterial and fungal communities was performed based on constrained multidimensional scaling using constrained analysis of principal components (CAP) from *vegan* (Oksanen et al., 2020). The significance of rootstock genotype, compartment, and soil status factors for each year of experiment used as constraint in the CAP was assessed *via* the permutation test from *vegan*. Bray–Curtis dissimilarity distances were also investigated using permutational multivariate analysis of variance (PERMANOVA) based on “adonis” function from *vegan* package (Anderson, 2001). Dissimilarities in the relative abundance of bacterial and fungal communities were visualized by network analysis of Bray-Curtis distances using the “make_network” and “plot_network” functions from the *phyloseq* package.

*MicrobiomeMarker* (version 1.1.1) was used for Limma-Voom method, using “run_limma_voom” function (α = 0.001) corrected with the False Discovery Rate (FDR), to discriminate microbial taxa above family between vineyard soil used for greenhouse experiment (Cao, 2020). To observe the contributions of vineyard and nursery microbiomes to root associated microbiome from the greenhouse experiment, the same package was used for linear discriminant analysis (LDA) effect size (LEfSe) method, using “run_lefse” function, to discriminate enriched microbial taxa above orders. This process was set with Kruskal (*α* = 0.001) and Wilcox (*α* = 0.001) tests, and corrected with the FDR.

### Visualization of bacterial endophytic taxa

Double labeling of oligonucleotide probes-Fluorescence *in situ* hybridization (DOPE-FISH) microscopy was carried out to visualize bacterial taxa within surface sterilized root samples, for the four conditions sampled in 2019. Fixation was carried out overnight at 4°C, in a paraformaldehyde solution (4% w/v in PBS pH 7.2) and rinsed three times with PBS. Samples were then treated with a lysozyme solution (1 mg ml^−1^ in PBS) for 10 min at 37°C, followed by dehydration in an ethanol series (25, 50, 75, and 99.9%; 15 min each step). DOPE-FISH was performed after cutting samples into small pieces, and then using probes from Eurofins (Germany) labelled at both 5′ and 3′ positions, summarized in Supplementary Table 3 (Bruez et al., 2020). A mixEUB (equivalent mixture of EUB338, EUB338II, EUB338III coupled with a Cy3 fluorochrome), a Chit probe specific to *Chitinophaga*, a Rhizo4 a probe specific to *Rhizobium* (16S), a Pseu22 probe specific to *Pseudomonas* from C3, C4, C5 clusters (16S), and a Pce probe specific to *Burkholderia* (23S), all coupled to Cy5 fluorochrome, were used. A NONEUB probe, coupled with Cy3 and Cy5, was also used independently as a negative control. Hybridization was performed at 46°C for all the probes except for the Pce which was done at 40°C, during 2 h 30 min, with 20 μl hybridization solution applied to each plant sample, placed on slides in a 50 ml moist chamber (also housing a piece of tissue imbibed with 5 ml of hybridization buffer). Each hybridization solution contained 20 mM Tris–HCl pH 8.0, 0.01% w/v SDS, 0.9 M NaCl, formamide at the concentration adapted for each probe: 15 ng μl^−1^ for a general probe, and 10 ng μl^−1^ for a specific probe. Post-hybridization was performed in 20 μl at 48°C (or 42°C for Pce) for 30 min with a post-FISH pre-warmed solution containing 20 mM Tris–HCl pH 8.0, 0.01% (w/v) SDS, 5 mM EDTA pH 8.0 and NaCl at a concentration corresponding to the formamide concentration used. Samples were rinsed with distilled water before being air-dried in the dark.

The samples were then observed under a confocal microscope (Olympus Fluoview FV1000 with multiline laser FV5-LAMAR-2 HeNe(G) and laser FV10-LAHEG230-2). X, Y, Z pictures were taken at 405, 488, 633 nm and with 20X objectives. Pictures were analyzed on imaris sofware. Pictures were cropped and whole pictures were sharpened. The light/contrast balance was also improved to better observe the image details, as seen when samples are observed in the dark under the microscope. Images shown in this publication represent the average of colonization.

## Results

### Initial matrix soil used for greenhouse experiment displayed different microbial communities

A total of 22,030,894 bacterial 16S rRNA gene sequences and 24,753,799 fungal ITS sequences were generated from the ninety-six samples (Supplementary Figure 1). After quality filtering, denoising, merging, chimera, and contaminant removing, 8,553,704 bacterial 16S rRNA gene sequences and 14,764,550 fungal ITS sequence remained and generated 31,096 bacterial and 7,994 fungal Amplicon Sequences Variants (ASVs). ASVs with less than 0.1% sequencing depth were removed.

**Figure 1:**
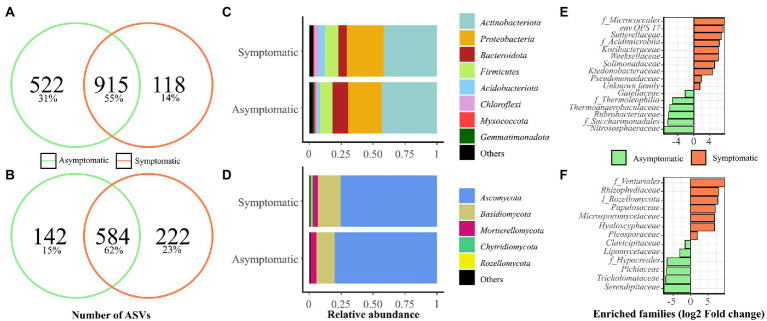
Microbial communities present in symptomatic (S) and asymptomatic (AS) soils from vineyard. Soil samples were collected in April 2019 and 2020 in each area (*n* = 6). Venn diagrams show the number of shared and specific ASVs for **(A)** bacterial, and **(B)** fungal communities. Abundances at phylum level are presented for **(C)** bacterial and **(D)** fungal communities. Phyla representing less than 1% of the total reads were grouped in “Others.” Enriched **(E)** bacterial and **(F)** fungal classes, orders, and families using Limma-Voom differential analysis (*p* < 0.001) corrected with FDR are shown.

First, 1,437 and 1,033 bacterial ASVs and 726 and 806 fungal ASVs were found in vineyard bulk soil samples from AS and S areas, respectively, regardless of the year of experiment (Figures 1A,B). The bacterial and fungal phyla showing more than 1% of average relative abundance are represented in Figures 1C,D, respectively. Bacterial phyla representing less than 1% abundance in AS and S soils, respectively, were grouped to “Others” and belong to *Desulfobacterota* (0.80, 0.75%), *Nitrospirota* (0.47, 0.27%), *Crenarchaeota* (0.38, 0.22%), *RCP2-54* (0.12, 0.33%) *Verrucomicrobiota* (0.11, 0.23%), *Bdellovibrionota* (0.12, 0.15%), *Patescibacteria* (0.12, 0%), and *Fibrobacterota* (0, 0.03%). The predominant fungal phyla are represented in Figure 1D with phyla from the “Others” group for AS and S soils, respectively, belonging to *Mucoromycota* (0.05, 0.15%) and *Glomeromycota* (0.02, 0%). Dominant bacterial classes for both years of the experiments in AS and S soils were, respectively, *Actinobacteria* (31.70, 34.99%), *Gammaproteobacteria* (14.96, 20.62%), *Alphaproteobacteria* (11.61, 8.76%), *Bacilli* (9.25, 9.49%), *Bacteroidia* (12.25, 6.45%), *Thermoleophilia* (8.85, 5.76%), and *Acidobacteriae* (2.11, 5.16%). The most represented fungal classes were *Sordariomycetes* (35.24, 36.64%), *Dothideomycetes* (19.19, 19.22%), *Tremellomycetes* (13.68, 15.87%), *Leotiomycetes* (15.08, 10.45%), *Eurotiomycetes* (6.77, 6.62%), and *Mortierellomycetes* (4.47, 4.77%).

Limma-Voom differential analysis was performed to obtain a better overview of the differences occurring between the two soils across the bacterial (Figure 1E) and fungal (Figure 1F) communities from the vineyard. This analysis revealed ten enriched bacterial groups in S soil (1.56 to 7.82 log2 fold change), mainly composed of *Proteobacteria* (*Sutterellaceae*, *Solimonadaceae*, *Pseudomonadaceae*) and *Bacteroidota* (*env.OPS 17*, *Weeksellaceae*), while AS soil was enriched with six groups (−2.17 to −7.57 log2 fold change) with a majority of *Actinobacteria* (*f_Thermophilia*, *Gaillellaceae*, *Rubrobacteriaceae*). In terms of fungi, seven enriched families were found in S soil (1.91 to 9.86 log2 fold change), accounting from a majority of *Ascomycota* (*Papulosaceae*, *f_Venturiales*, *Hyaloscyphaceae*, *Pleosporaceae*), while six enriched families were detected in AS soil (−1.50 to −7.76 log2 fold change) mainly belonging to *Ascomycota* (*f_Hypocreales*, *Clavicipitaceae*, *Lipomycetaceae*, *Pichiaceae*).

In vineyard soils, bacterial richness was slightly lower in S area compared with AS area and bacterial α- and β-diversity indices were affected by soil status independently of the year of experiment (Table 1; Supplementary Table 4). Fungal richness and α-diversity were not significantly different between the two soils and these indices were mainly affected by the year of experiment. The fungal β-diversity was significantly different between the two soils. These results indicate a difference in the structure and composition of the microbial community between S and AS soils as previously suggested in Darriaut et al. (2021).

**Table 1:** Effect of soil type (S, AS), rootstock genotype (RGM, 1103P), and year of the experiment (2019, 2020) on richness, α-diversity, and β-diversity of bacterial and fungal communities in the bulk, rhizosphere, and roots compartments in the greenhouse experiments.

			Richness (Observed ASVs)		α-diversity (Simpson’s index)		β-diversity (Bray-Curtis)		
			*F*	*P*	*F*	*P*	*F*	*R* ^2^	*P*
Bacteria	Vineyard	Soil	1.147	0.312	4.553	**0.041**	34.203	0.490	**0.001**
		Year	13.841	**0.005**	0.308	0.592	21.993	0.315	**0.001**
	Bulk	Soil (S)	0.860	0.364	0.551	0.467	9.693	0.238	**0.001**
		Genotype (G)	0.043	0.837	1.374	0.256	1.210	0.029	0.259
		Year	0.253	0.620	2.406	0.137	9.151	0.225	**0.001**
		S × G	5.031	**0.037**	0.560	0.463	1.595	0.039	0.129
	Rhizosphere	Soil	1.045	0.319	2.961	0.101	14.437	0.308	**0.001**
		Genotype	0.021	0.885	0.042	0.841	1.118	0.024	0.273
		Year	4.629	**0.045**	10.011	**0.005**	11.460	0.245	**0.001**
		S × G	0.399	0.535	0.001	0.977	0.798	0.017	0.521
	Root	Soil	1.648	0.215	0.001	0.996	2.632	0.085	**0.001**
		Genotype	6.866	**0.017**	1.577	0.224	2.132	0.069	**0.003**
		Year	0.808	0.380	1.440	0.245	5.813	0.189	**0.001**
		S × G	0.086	0.773	2.610	0.123	1.232	0.040	0.192
Fungi	Vineyard	Soil	0.797	0.395	8.273	0.018	16.974	0.351	**0.001**
		Year	144.183	**<0.001**	62.261	**<0.001**	16.887	0.349	**0.001**
	Bulk	Soil	1.328	0.263	0.684	0.419	10.648	0.226	**0.001**
		Genotype	0.922	0.350	2.561	0.126	0.947	0.020	0.403
		Year	14.951	**0.001**	15.109	**<0.001**	15.623	0.331	**0.001**
		S × G	2.047	0.169	1.082	0.311	0.908	0.019	0.442
	Rhizosphere	Soil	0.222	0.643	0.104	0.751	11.822	0.254	**0.001**
		Genotype	0.015	0.904	0.014	0.907	1.580	0.033	0.132
		Year	5.280	**0.033**	7.127	**0.015**	12.901	0.278	**0.001**
		S × G	1.372	0.256	0.058	0.812	1.172	0.025	0.291
	Root	Soil	3.804	0.066	15.161	**<0.001**	1.868	0.065	**0.010**
		Genotype	5.058	**0.037**	6.518	**0.019**	2.557	0.089	**0.001**
		Year	130.83	**<0.001**	6.401	**0.020**	4.279	0.149	**0.001**
		S × G	0.396	0.537	0.771	0.391	1.083	0.038	0.306

Significances were assessed through a Type II ANOVA for richness and α-diversity while PERMANOVA was used for β-diversity. *p*-values <0.05 are highlighted in bold.

### Microbial community structure assessed using metabarcoding and MALDI-TOF-MS of cultivable isolates showed differences depending on soil composition and rootstock genotype

**Figure 2:**
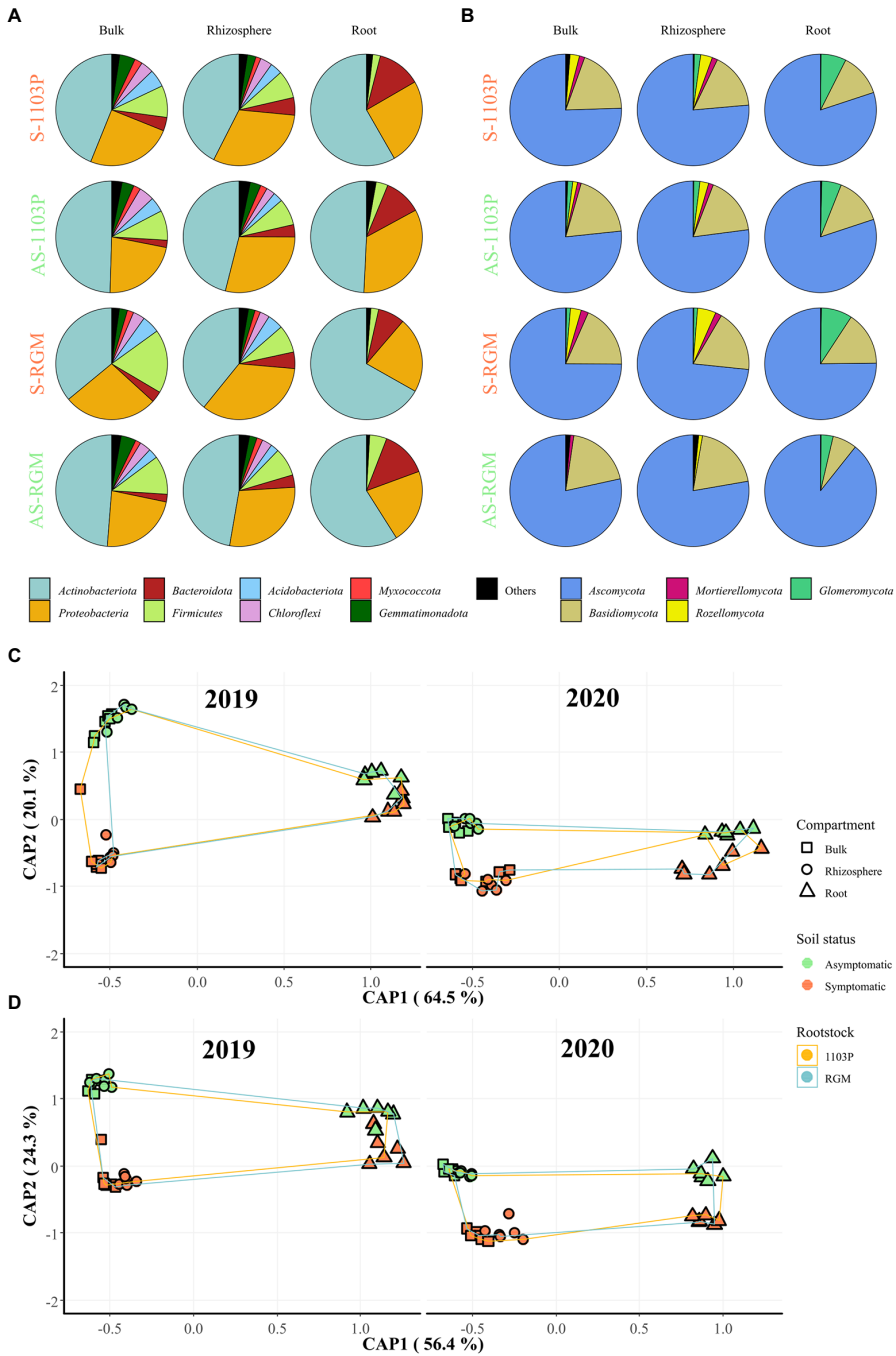
Bacterial and fungal communities associated with grapevine rootstocks root systems compartments and bulk soils after 4.5 months of growth in S and AS soils in the greenhouse. CS/1103P and CS/RGM grafted plants were grown either on symptomatic (S-1103P and S-RGM samples, respectively) and asymptomatic vineyard soils (AS-1103P and AS-RGM samples, respectively). The relative abundance at the phylum level in each compartment is represented for **(A)** bacterial and **(B)** fungal communities, regardless of year of experiment. Phyla accounting for less than 1% of the total abundance in communities were grouped in “Others.” Constrained analysis of principal coordinates (CAP) of samples by compartment related to CS plants grafted on 1103P and RGM rootstocks grown in symptomatic and asymptomatic soils in greenhouse, is shown for 2019 and 2020 experiments for **(C)** bacterial and **(D)** fungal communities.

After 4 months of greenhouse cultivation, bacterial communities in bulk soil in the pots, rhizospheres and plant roots were largely composed of *Actinobacteria*, *Proteobacteria*, *Bacteroidetes*, and *Firmicutes* (Figure 2A), while *Ascomycota* and *Basidiomycota* were predominant in the fungal division (Figure 2B). Constrained analysis of principal coordinates (CAP) based on the two rootstock genotypes, two soils, and three compartments, displayed for both rootstocks that bulk and rhizosphere clustered together, distinctly to the roots, and were grouped depending on the soil status for bacterial (Figure 2C) and fungal communities (Figure 2D). Even if an effect linked to the year of the experiment can be observed in particular for all fungal richness and diversity indices (Table 1), the repartition of the samples was quite similar in 2020 compared to 2019 (Figures 2C,D). No clear difference was observed between bulk soil in pots and rhizosphere for any of these indices, regardless of the rootstock or soil status. The lowest richness and Simpson’s indices were observed in root samples (Figures 2A,B; Supplementary Table 4) for bacteria and fungi. While richness was not significantly different between root conditions, bacterial and fungal Simpson’s indices varied between root samples. Bacterial Simpson’s index appeared slightly lower in roots of RGM in S soils; and fungal Simpson’s index was higher in roots in S soils compared to those in AS soils for both rootstocks. The soil status significantly impacted the β-diversity of both bacterial and fungal communities in every compartment, while only the fungal Simpson’s diversity in roots was driven by soil composition. This difference between AS and S soils was more pronounced in the bulk and rhizosphere compartments than in the roots for both microbial communities (Table 1). Interestingly, genetic background of the rootstock significantly affected both bacterial and fungal richness and β-diversity, as well as fungal α-diversity exclusively in the roots (Table 1). Furthermore, PERMANOVA revealed that the most influential factor for both bacterial and fungal communities was the compartment (i.e., rhizosphere, bulk soil, and roots), while soil status and year of experiment had similar effects (Table 2).

**Table 2:** Effect of soil type (S, AS), compartment (bulk, rhizosphere, and roots), rootstock genotype (1103P and RGM), and year of the experiment (2019, 2020) on richness, α-diversity, and β-diversity of bacterial and fungal communities in the dataset from greenhouse experiments.

		Richness (Observed ASVs)		α-diversity (Simpson’s index)		β-diversity (Bray-Curtis)	
		*F*	*P*	*F*	*P*	*F*	*P*
Bacteria	Soil	0.454	0.503	0.002	0.967	11.65	**0.001**
	Compartment	120.31	**<0.001**	55.512	**<0.001**	24.25	**0.001**
	Rootstock	1.441	0.234	1.793	0.185	1.44	0.144
	Year	0.423	0.517	1.420	0.238	10.72	**0.001**
Fungi	Soil	2.996	0.088	8.892	**0.004**	11.60	**0.001**
	Compartment	224.196	**<0.001**	28.604	**<0.001**	17.13	**0.001**
	Rootstock	1.717	0.194	5.259	**0.025**	1.77	0.068
	Year	44.178	**<0.001**	24.148	**<0.001**	11.78	**0.001**

Significances were assessed through a Type II ANOVA for richness and α-diversity while PERMANOVA (permutations = 999) was calculated by terms from “capscale” function using Soil + Compartment + Rootstock + Year model. *p*-values <0.05 are highlighted in bold.

**Figure 3:**
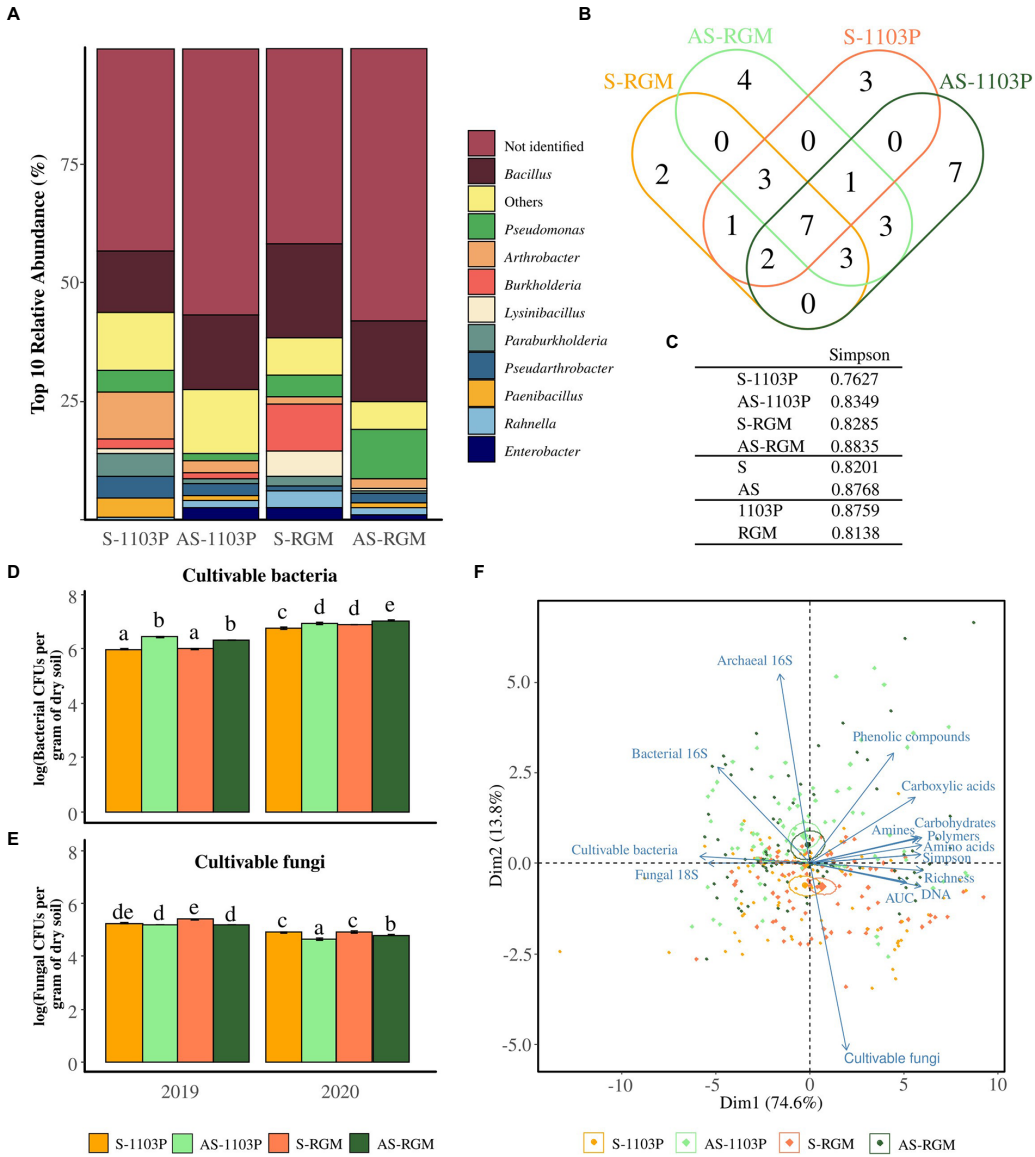
Microbial profile using cultivable-based approaches and q-PCR in the rhizosphere of grapevine plants after 4.5 months of growth in S and AS soils in the greenhouse. Diversity of cultivable bacteria isolated from rhizosphere and identified among the 4 conditions through MALDI-TOF-MS, demonstrated by **(A)** the relative abundance of the top 10 taxa at the genus level, **(B)** the Venn diagram illustrating the overlap of the genera, and **(C)** the associated Simpson’s diversity index. Histograms representing the level of populations of cultivable **(D)** bacteria and **(E)** fungi for 2019 and 2020 experiments. Bars represent means ± SE (*n* = 3), different letters indicate significantly different means according to pairwise comparisons based on Student t tests (*p* < 0.05). **(F)** Ordination biplot of principal component analysis (PCA) for level of cultivable microorganisms, Eco-Plates measurements (Area Under AWCD Curve = AUC, Simpson’s diversity, functional richness, and the families of consumed substrates), the total DNA extracted from the rhizosphere (*n* = 5) and the amplicons (fungal 18S rRNA gene, archaeal and bacterial 16S rRNA genes).

Two hundred bacterial isolates from rhizosphere samples for each condition (i.e., AS-1103P, S-1103P, AS-RGM, and S-RGM) were then analyzed on MALDI-TOF-MS. Among these 800 isolates, 401 (50%) mass profiles matched the Biotyper database with score values >1.8. One hundred sixty-nine isolates from asymptomatic soils were identified and 230 isolates from S soils. Score values above 1.8 matched for 36 genera, while score values above 2.0 matched for 83 species. The isolated bacteria were predominantly members of the genus *Bacillus* (16.6%), followed by *Pseudomonas* (5.25%), *Arthrobacter* (4%), and *Burkholderia* (3.4%). Genera detected less frequently were categorized in the “Others” group (Figure 3A) and correspond to *Ralstonia*, *Buttiauxella*, *Variovorax*, *Paenarthrobacter*, *Rhizobium*, *Streptomyces*, *Flavobacterium*, *Peanibacillus*, *Dyella*, *Serratia*, *Caballeronia*, *Brevibacillus*, *Microbacterium*, *Sphingomonas*, *Falsibacillus*, *Staphylococcus*, *Acinetobacter*, *Amicolaptosis*, *Aquincola*, *Brachybacterium*, *Cupriavidus*, *Gordonia*, *Herbaspirillum*, *Leifsonia*, *Rhodococcus*, and *Sinomonas*. Certain genera were identified in only one condition (Figure 3B), with one genus being common to the S soils (*Rhizobium*) and three genera being common to the AS soils (*Peanibacillus*, *Brevibacillus*, *Buttiauxella*). The Simpson’s indices generated from these isolates are shown (Figure 3C) and even though the differences could not be supported by statistical tests, we can see that this index is higher among the conditions in AS soils compared to S soils, and lower in the RGM compared to rootstock 1103P (Figure 3C).

### Microbial activity and level of cultivable population in the rhizosphere differed according to the soil composition

Cultivable bacteria associated with the rhizosphere soil of the RGM and 1103P rootstocks grown in S and AS soils during both years of sampling ranged from 10^5^ to 10^8^ CFUs/g (Figure 3D), while cultivable fungi ranged from 10^4^ to 10^7^ CFUs/g (Figure 3E). Plating methods revealed that the level of cultivable bacteria was significantly different among the four conditions, in 2019 [*F*(3,8) = 113.1, *p <* 0.001], and in 2020 [*F*(3,8) = 15.67, *p* < 0.001; Figure 3D] with a higher level observed in the AS soil compared to the S one for both rootstocks. In parallel, significant differences were also observed for the level of cultivable fungi in 2019 [*F*(3,8) = 34.97, *p* < 0.001], and in 2020 [*χ*^2^ = 27.578, ddl = 3, *p* < 0.001] but with a lower level observed in the AS soil compared to the S one for both rootstocks (Figure 3E). The abundance of cultivable bacteria and fungi was then significantly affected by the year of the experimentation, the soil status and the rootstock genotype (Table 3). The estimation of microbial biomass using q-PCR revealed significantly higher level of archaeal and bacterial amplicons in AS rhizosphere compared to S rhizosphere for both rootstocks during the 2 years of sampling, while no differences were detected for fungal amplicons (data not shown and Table 3).

**Table 3:** Effect of soil type (S, AS), rootstock genotype (1103P, RGM) and year of the experiment (2019, 2020) on the rhizosphere microbial community.

		Rootstock		Soil		Year	
		*F*	*P*	*F*	*P*	*F*	*P*
	Cultivable bacteria	11.68	**0.001**	43.61	**<0.001**	403.93	**<0.001**
	Cultivable fungi	8.629	**0.004**	49.603	**<0.001**	164.001	**<0.001**
Biolog™ System	Area under curve	12.33	**0.002**	48.59	**<0.001**	781.71	**<0.001**
	Simpson’s evenness	0.178	0.678	0.030	0.865	201.371	**<0.001**
	Functional richness	0.024	0.880	8.489	**0.009**	158.114	**<0.001**
	Amines	0.554	0.466	1.330	0.263	98.976	**<0.001**
	Amino acids	1.529	0.231	15.655	**<0.001**	273.105	**<0.001**
	Carbohydrates	0.015	0.903	14.349	**<0.001**	563.578	**<0.001**
	Carboxylic acids	0.022	0.884	9.353	**0.006**	122.215	**<0.001**
	Phenolic compounds	1.689	0.209	2.157	0.158	137.548	**<0.001**
	Polymers	1.043	0.320	3.951	0.061	73.700	**<0.001**
qPCR	DNA	0.189	0.666	1.295	0.260	179.021	**<0.001**
	Bacterial 16S rRNA gene	2.056	0.157	21.367	**<0.001**	122.098	**<0.001**
	Archaeal 16S rRNA gene	0.375	0.543	41.456	**<0.001**	39.956	**<0.001**
	Fungal 18S rRNA gene	0.239	0.627	1.016	0.318	86.702	**<0.001**

Microbial level of cultivable populations, the Biolog system parameters, the microbial DNA, and the q-PCR measurements in the rhizosphere samples are presented. Significances were assessed through a Type II ANOVA. *p*-values <0.05 are highlighted in bold.

Microbes of both soils showed the same activities in Eco-Plates measurements, but with different intensities (Supplementary Table 5). The microbial activities represented by the AUCs from the Biolog Eco-Plates™ technology were significantly higher for S soils compared to AS ones for both rootstocks in 2019 [*F*(3,8) = 25.25, *p* < 0.001], and 2020 [*F*(3,8) = 34.1, *p* < 0.001].

The biplot PCA for q-PCR, level of cultivable microbes, and q-PCR measurements in the rhizosphere revealed two overlaps of confidence intervals between the S-1103P and S-RGM conditions, as well between AS-1103P and AS-RGM (Figure 3F). Dimensions (Dim1 and Dim2) accounted for 88.4% of total variance. Symptomatic feature was mostly found in the negative side of Dim2, which was correlated with the level of population of cultivable fungi, the total extracted DNA, the general activities, and richness measured in Eco-Plates (i.e., AUC, richness, respectively; Table 3). On the other hand, asymptomatic samples were mainly found in the positive side of Dim2, which correlated with all other measurements, including the cultivable bacteria population, number of bacterial and archaeal 16S rRNA genes, and all the other Eco-Plates measurements (i.e., Simpson’s diversity, amino acids, polymers, carbohydrates, amines, carboxylic acids, and phenolic compounds, Table 3).

### Soil status slightly influenced grapevine growth in greenhouse experiments

Phenotypic measurements were taken at different times during the experiments (data not shown) and after 4.5 months of growth. Data presented in Supplementary Table 6 show that the growth of the plants was higher in 2020 than in 2019. Interestingly, although no effect of soil status on plant growth parameters or chlorophyll content was observed in 2020, a higher stem length and chlorophyll content was measured for plants grown in AS soils compared to S soils in 2019. Shoot dry weight was significantly lower in plants grown in S soils compared to AS soils only for CS/1103P in 2019.

### Vineyard and nursery microbiomes contribute to root associated microbiome in the greenhouse experiment

We compared the microbial diversity and richness of the soil right after sampling in the vineyard and the bulk soil harvested in the pots after the greenhouse experiment (Supplementary Figure 2A). The bacterial richness was more affected while its diversity was globally similar between vineyard and greenhouse for a same soil status (S or AS). At the opposite, for fungal communities the richness was less impacted than the diversity. In addition, CAP based on the bulk soil from the greenhouse experiment and bulk soil collected in the vineyard demonstrated clear differences in bacterial (Supplementary Figure 2B) and fungal communities (Supplementary Figure 2C), mainly due to soil status for both sampling years. Specific genera were enriched in both investigated vineyard and greenhouse bulk soils, regardless of sampling year (Supplementary Figure 2B), with higher number of bacterial and fungal taxa enriched in vineyards (36 and 24, respectively) compared to greenhouse (7 and 13, respectively).

**Figure 4:**
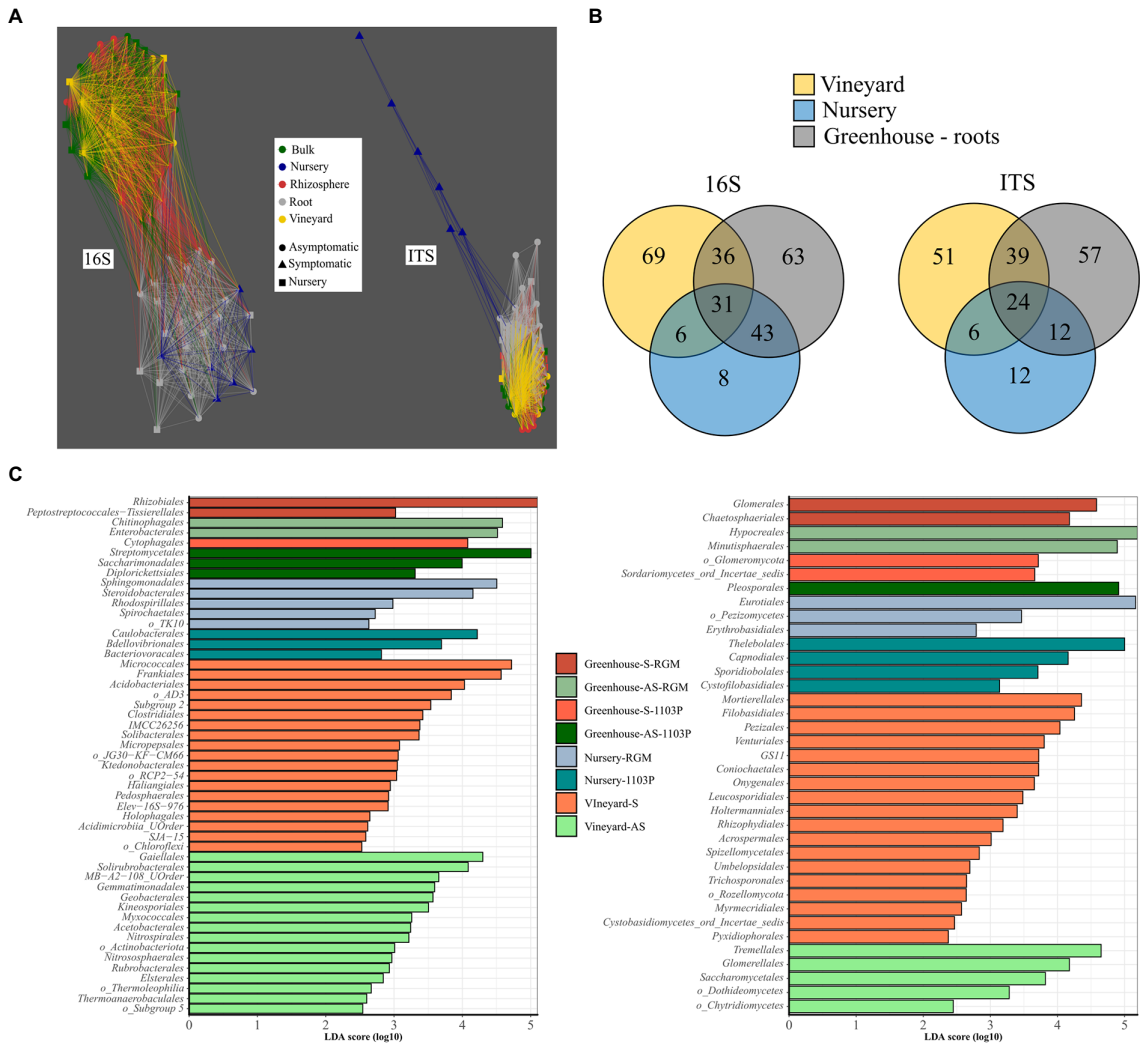
The contribution of vineyard and nursery microbiomes to root associated microbiome in the greenhouse disposal. **(A)** Network analysis of bacterial (16S rRNA gene) and fungal (ITS) taxa, in terms of relative abundance, in vineyard soils, roots from nursery plants before planting, and greenhouse compartments (i.e., bulk, rhizosphere, root), using Bray–Curtis distances less than 0.95. **(B)** Venn diagram presenting the number of shared and specific bacterial and fungal genera in vineyard soils, nursery plants, and root compartment from the greenhouse disposal. **(C)** LEfSe displaying the enriched orders in vineyard soils (symptomatic (S) and asymptomatic A(S)), nursery plant roots (1103P and RGM rootstocks grafted with CS), and root compartments from greenhouse experiment in each condition (AS-1103P, S-1103P, AS-RGM, S-RGM). Bacteria are in the left and fungi on the right.

Network analysis of Bray-Curtis distances clustered roots from greenhouse plants and roots from nursery plants and separated them from another cluster composed of bulk, rhizosphere, and vineyard soil compartments for bacterial communities (Figure 4A). The same analysis shows the fungal communities of roots from nursery plants were clearly different from those of all other compartments (Figure 4A).

Around 12% of the bacterial and fungal genera (31 and 24 genera, respectively) found in vineyard soils, nursery plant roots before plantation and greenhouse plant roots were common for all these compartments (Figure 4B). Moreover, 3 and 6% of the bacterial (i.e., *Nordella*, *Paenisporosarcina*, *Allokutzneria*, *Salinispira*, *Phaselicystis*, *Peredibacter*, *FFCH7168*, *SWB02*) and fungal (i.e., mainly *Ascomycota* from *Ramularia Debaryomyces*, *Neosetophoma*, *Botrytis*, *Vermiconia*, *Microdochium, Zymoseptoria*) genera, respectively, were found exclusively in nursery samples and were not detected in greenhouse root samples (Figure 4B). Among the genera found in the roots at the end of the experiment, 25 and 9% of bacterial and fungal genera, respectively, were also found only in roots of nursery plants (i.e., before planting). Enriched taxa were largely found in vineyard soils, accounting for 35 bacterial and 23 fungal orders, while only 8 and 7, respectively, were found in nursery soils (Figure 4C).

**Figure 5:**
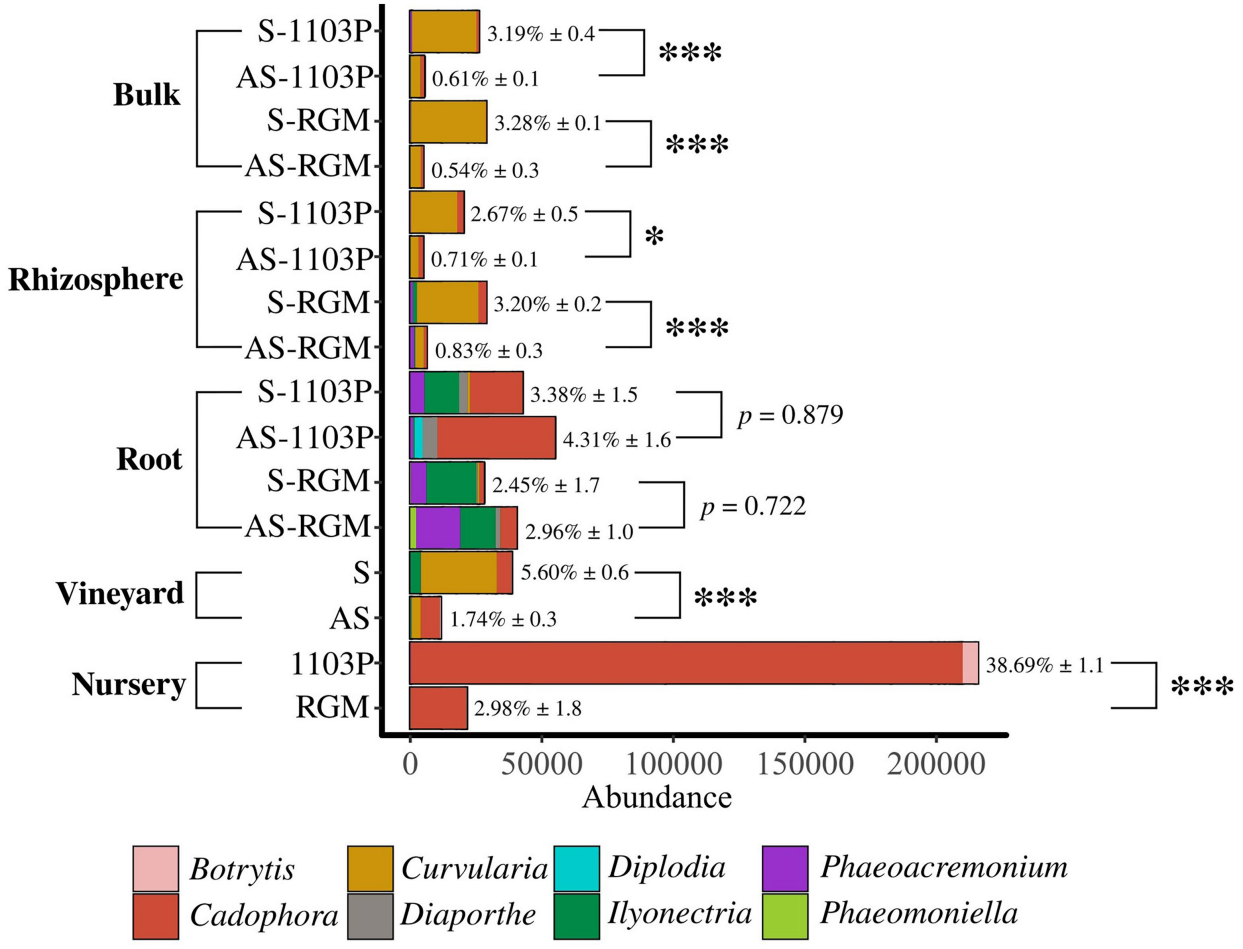
Abundance of fungal genera associated to grapevine diseases in the different compartments related to greenhouse experiments. Roots from nursery plants before plantation, S and AS soils from vineyard before the greenhouse experiments, and bulk soils, rhizosphere and roots of 1103P and RGM rootstocks grafted with CS after 4.5 months of growth either in S or AS soils. Percentages (± SE) indicate proportions of sequences affiliated with pathogenic fungi relative to total sequences. Significant differences (*p < 0.05; ***p < 0.001) between compared conditions were detected with Student t or Wilcoxon tests.

In addition, certain fungal genera associated with known grapevine diseases listed in Supplementary Table 7, were detected across the samples belonging to *Botrytis*, *Cadophora*, *Curvularia*, *Diaporthe*, *Diplodia*, *Ilyonectria*, *Phaeoacremonium*, and *Phaeomoniella* (Figure 5). Overall, they revealed a higher abundance in symptomatic initial soil compared to the asymptomatic condition in the vineyard [Student *t* (7.541) = 5.575, *p* < 0.001], but also in bulk soil [Student *t* (13.27) = 10.205, *p* < 0.001] and rhizosphere [Student *t* (17.571) = 6.472, *p* < 0.001] samples in the greenhouse during the two experiments. No significant differences were found for root samples during the greenhouse experiment. Some differences in fungal genera composition were found. In soil and rhizosphere samples, we primarily detected *Curvularia* and *Cadophora*. *Ilyonectria* was identified in higher abundance in S vineyard soil and, interestingly, in S-RGM rhizosphere samples. RGM rhizosphere samples appeared enriched in *Phaeoacremomium* and *Ilyonectria* genera compared to 1103P samples, and this difference seems to be also detected in root samples. Surprisingly, a very high abundance of *Cadophora* and *Botrytis* genera was found in roots of CS/1103P nursery plants compared to CS/RGM ones [*F*(2,712) = 14.092, *p <* 0.001; Figure 5].

### Visualization of endophytic microorganisms associated to roots

Based on ITS sequencing, the phylum *Glomeromycota* was globally enriched in roots (6.38%) compared to bulk [0.98%; Student *t* (46) = 4.155, *p* < 0.001] and rhizosphere [1.30%; Student *t* (46) = 3.939, *p* < 0.001] compartments across the four conditions (Figure 2B), with higher amounts in S (RGM: 8.98%, 1103P: 7.31%) compared to AS (RGM: 3.50%, 1103P: 5.68%) roots for RGM and 1103P rootstocks, independently of the year of experiment. The mycorrhizal colonization of the four conditions was investigated at the final point of sampling in the second experiment using staining and microscopy methods (Supplementary Figure 3). The mycorrhizal frequency (F) was not significantly different among the conditions (*χ*^2^ = 5.9862, ddl = 3, *p* = 0.1123; Supplementary Table 8). However, the intensity of the mycorrhizal colonization (M) was significantly different [*F*(3,16) = 5.313, *p* = 0.001]. Interestingly, RGM rootstock showed a significantly higher intensity of colonization when grown in S soils compared to AS soils, while no significant difference was observed for the 1103P rootstock. This result is consistent with the ITS-based sequencing analysis since a significantly higher relative abundance of *Glomeromycota* sequences was found in S-RGM roots compared to the other samples (Supplementary Table 8).

**Figure 6:**
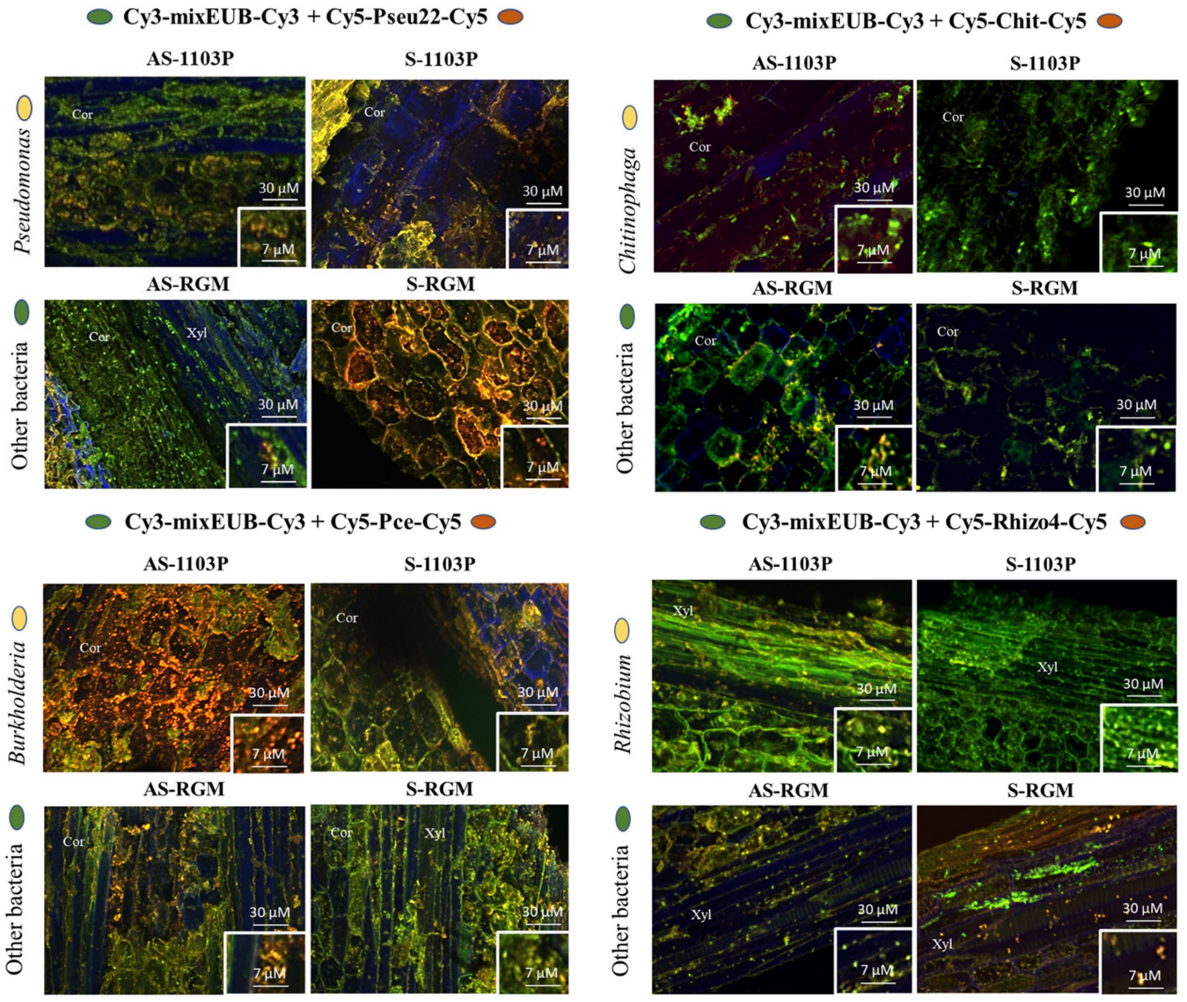
Microphotographies of the root colonization by *Chitinophaga*, *Rhizobium*, *Burkholderia*, and *Pseudomonas* genera using DOPE-FISH microscopy across the different conditions within roots sampled during the 2019 greenhouse experiment. Cor = cortex, Xyl = xylem.

Four bacterial genera, *Pseudomonas*, *Chitinophaga*, *Burkholderia* (*Caballeronia*-*Paraburkholderia*), and *Rhizobium* (*Allorhizobium*-*Neorhizobium*-*Pararhizobium*), were targeted for DOPE-FISH microscopy to confirm their presence and localization within the root tissues. These four genera belong to the top ten bacterial genera identified in root samples of both genotypes at the end of the 2019 greenhouse experiment (Supplementary Figure 4). They were chosen according to several criteria, such as their high relative abundance in the samples, the putative biological functions of some members of these genera and the methological possibility to target them in DOPE-FISH experiments. They were all visualized in the root endosphere compartment of both RGM and 1103P rootstocks in either cortex or xylem zones (Figure 6). Naturally autofluorescent microbes were slightly detected in root endosphere using negative NONEUB probe, confirming the specificity of the probes used to target microorganisms (Supplementary Figure 5).

## Discussion

In plants, as in humans, many physiological and pathological diseases are linked to an imbalance of the microbiota, also called dysbiosis. This imbalance is caused by a variety of factors, and restoring the equilibrium requires both a precise diagnosis and identification of the sources of potential variability that can influence the microbiota. In a previous study, we focused on vine declines unrelated to symptoms of pathological diseases. In each vineyard studied, the slight differences detected in soil chemical composition between symptomatic and asymptomatic areas could not be explained by differences in vine growth (lower vigor, measured as the weight of pruned wood in the winter, and higher mortality of the plants) (Darriaut et al., 2021). In these four vineyards, microbial enzymatic activities were lower in symptomatic soils compared to asymptomatic soils, suggesting a microbial dysbiosis in areas subjected to decline. Year of sampling was pointed out as a major factor influencing the diversity and composition of the microbiota (Berlanas et al., 2019; Cureau et al., 2021). We observed here that while microbial communities evolved from year to year, the differences in microbial composition between S and AS soils was confirmed over the 2 years of sampling, with more putative fungal pathogens in S soils. We performed a greenhouse experiment with these two soils to understand the impact of the microbial composition of the soil on the development of the vines and on the root-associated microbiome, but also the effect of the rootstock on these responses.

We first observed that the bulk soil in pots at the end of the greenhouse experiment was different from the initial bulk soil from the vineyard in terms of microbial diversity and richness. Soil microorganisms have complex interrelationships within natural soils, and their transposition into greenhouse experiment could modify the structure of microbial communities. For fungal communities in our experiment, Simpson’s index was impacted rather than richness, suggesting that their sensitivity to environmental change is greater than for bacterial communities. Moreover, a higher number of bacterial and fungal taxa were enriched in the bulk soil from the vineyard compared to the soil from the greenhouse. A reduced diversity or altered community structure is often expected in the pot experiment compared to soils in their natural system (Berg et al., 2016), which was partly true in terms of the Simpson’s index of both bacterial (Symptomatic soils during 2020) and fungal (Asymptomatic soils during 2019) communities. Pots kept in the greenhouse for long-term cultivation are usually supplied with nutrient solution or are potted with plant substrate, with both, combined or alone, drastically diminishing the microbial richness and diversity (Zachow et al., 2014; Granzow et al., 2017; Wang et al., 2017). Herein, the pots in the greenhouse were watered daily without adding any nutrients, which affected the fungal diversity. Kaisermann et al. (2015) demonstrated through small fluctuations of soil water content that fungal communities were largely affected, unlike bacterial communities, which were less sensitive to these small environmental constraints. Other different environmental conditions between the vineyard and pots could have affected the microbial composition of the soil, such as temperature, soil compaction, oxygenation (Cookson et al., 2007; Rousk et al., 2010; Brockett et al., 2012). Even so, the initial difference in the composition of microbiomes associated with symptomatic and asymptomatic vineyard soils persisted during the greenhouse experiment with microbial communities affected differently.

After 4 months of the greenhouse experiment, microbial communities were mainly driven by the compartment studied (i.e., bulk soil, rhizosphere and roots). The differences of microbiota between the soil-rootstock-scion continuum have been previously identified in grapevines (Compant et al., 2011; Zarraonaindia et al., 2015; Deyett and Rolshausen, 2020; Liu and Howell, 2021; Swift et al., 2021). Plant compartments provide specific microbial niches leading to distinct microbiome associations and functionalities (Rossmann et al., 2017). The microbial diversity is usually lower with a higher degree of specialization proximal to the roots (Bonito et al., 2014). Several works also highlighted the decrease of bacterial and fungal richness and diversity metrics from soil to roots (Marasco et al., 2013; Samad et al., 2017; D’Amico et al., 2018; Marasco et al., 2018; Martínez-Diz et al., 2019; Carbone et al., 2021; Marasco et al., 2022).

Despite the use of different primers for 16S rRNA gene amplicon sequencing between the studies, the bacterial communities found in our samples were similar to previous findings, with a predominant relative richness of *Actinobacteria*, *Proteobacteria*, *Bacteroidota*, *Firmicutes*, *Acidobacteria*, *Chloroflexi*, *Myxococcota*, and *Gemmatimonadota* (Samad et al., 2017; Berlanas et al., 2019; Liang et al., 2019; Deyett and Rolshausen, 2020; Dries et al., 2021a; Swift et al., 2021). Most of these phyla represent the core microbiome of vineyard topsoils identified through a global microbiome survey within 200 vineyards collected in 13 countries (Gobbi et al., 2022). Samad et al. (2017) found that lower diversity was recovered by cultivation from rhizosphere samples compared with community sequencing. However, in the present study, genera belonging to the most abundant phyla (*Proteobacteria*, *Actinobacteria* and *Firmicutes*) were recovered by cultivation and sequencing of the strains. These cultivation-based approaches are currently being improved thanks to the identification of culture medium, which allows more bacteria to grow *in vitro*, derived, for example, from cell extracts (Sarhan et al., 2019). The availability of the strains will allow further functional analysis of their characteristics related to plant growth promotion, stress tolerance and biocontrol properties (Dries et al., 2021b; Bettenfeld et al., 2021; Darriaut et al., 2022). The structure of root bacterial communities was distinct to the ones found in the rhizosphere and bulk soil, as reported in previous studies (Zarraonaindia et al., 2015; Samad et al., 2017; D’Amico et al., 2018; Swift et al., 2021; Marasco et al., 2022). Overall, bacterial α-diversity was only significantly driven by the compartment factor, confirming the roots as a selective barrier for large panel of bacterial taxa. The presence of the most represented endophyte bacterial genera (i.e., *Pseudomonas*, *Chitinophaga*, *Rhizobium*, and *Burkholderia*) among the four conditions was confirmed using DOPE-FISH microscopy.

Identification of fungal communities by amplicon sequencing is generally based on a region of the ITS, although this methodology has been described as lacking information in some cases due to high hypervariability (Kiss, 2012; Vu et al., 2018). Here, two libraries were used based on both fungal ITS1 and ITS2 regions, the latter being created with two primer mixes by a nested PCR approach. Indeed, the fungal ITS2 barcoding region was found to recover more DNA sequences for fungal analysis than LSU, SSU, or even ITS1 (Schoch et al., 2012), and remains the favorite molecular marker to study fungal communities (Tedersoo et al., 2014). The detected fungal communities were dominantly composed of *Ascomycota* and *Basidiomycota* in bulk soil, rhizosphere, and root endosphere, as reported in previous studies (Berlanas et al., 2019; Martínez-Diz et al., 2019; Deyett and Rolshausen, 2020; Carbone et al., 2021; Liu and Howell, 2021; Swift et al., 2021; Zahid et al., 2021; Marasco et al., 2022). Network analysis of Bray-Curtis distances distinguished root from soil samples. This network is consistent with the results from Zarraonaindia et al. (2015) and Marasco et al. (2018), which found distinct clusters and connections from the soil × root samples. However, a different pattern was found for the fungal communities with a clustering of the greenhouse and vineyard samples, probably due to the strong segregation of nursery samples. While fungal richness was significantly influenced by the year of experiment and compartment, the fungal diversity was mostly affected by compartmentalization in addition to soil status, rootstock genotype, and year of experiment. Fungal communities are known to be distinct in diversity, composition, and functionality in the different grapevine-associated compartments (Carbone et al., 2021; Swift et al., 2021; Marasco et al., 2022). In the present study, the dissimilarities were predominant in the root-associated microbiome, as it was demonstrated for bacterial communities.

Most studies on the microbial community of the vine in a context of decline focused on GTDs and the composition of the microbial community in the wood (Bruez et al., 2016, 2020; Bettenfeld et al., 2021; Fotios et al., 2021; Haidar et al., 2021). The rhizosphere and root endosphere microbial communities had not yet been investigated when the observed decline was not associated with symptoms of pathologies or mineral deficiencies. Given that the microbial structure of the soil is a key component of plant health, it is important to define the characteristics of a balanced underground microbiome. The composition of the microbial communities in the bulk soil and rhizosphere was clearly divergent between samples from S and AS conditions. Soil status also influenced the root communities, especially for fungi. Significant differences in microbial activities measured using Eco-Plates were identified between the rhizosphere samples, confirming that these communities might have different functions. Although no symptoms of disease were detected on plants within the two areas in the vineyard and in the greenhouse, a higher relative abundance of putatively pathogenic fungi (mainly *Cadophora*, *Curvularia* and *Ilyonectria* genera) was found in S bulk soil and rhizosphere samples than in AS samples. Among *Cadophora* genus, species were identified as *C. luteo-olivacea* (Navarrete et al., 2011), *C. malorum* (Travadon et al., 2015), and *C. melinii* (Gramaje et al., 2011) that are associated to Petri disease. Some of the detected phytopathogens were more abundant in roots, as for example *Ilyonectria* or *Phaeoacremonium*, than in soil compartments, which may not be surprising since these genera are affiliated with GTDs (Lade et al., 2022).

The role of nurseries and propagation process as a primary source of GTDs inoculum has been previously documented (Aroca et al., 2010; Gramaje and Armengol, 2011; Nerva et al., 2019; Gramaje et al., 2022; Lade et al., 2022). Special care is taken during the grafting and propagation processes to limit the presence of GTDs related to fungi and viruses. In our study, the fungal community of roots from the nursery was highly divergent to that of the roots at the end of the greenhouse experiment. A higher abundance of *Cadophora* and *Botrytis* (responsible of grey mold disease), was found in the roots of CS/1103P nursery plants in particular. This probably explains the higher abundance of *Cadophora* in greenhouse roots of CS/1103P. Several mechanisms to control *Botrytis* in grapevines were described. These were mediated by endophytic bacteria, such as *Streptomycetes*, *Pseudomonas*, *Bacillus*, *Acinetobacter*, *Burkholderia*, *Erwinia*, *Pantoea agglomerans*, or *Micromonospora* (Compant et al., 2013). Some of these potentially antagonistic genera (i.e., *Pseudomonas*, *Streptomyces*, *Acinetobacter*, *Burkholderia*) were found in rhizosphere samples among the four conditions (i.e., AS-1103P, S-1103P, AS-RGM, S-RGM) using MALDI-TOF-MS, as well as in root samples using DOPE-FISH microscopy (i.e., *Burkholderia*, *Pseudomonas*) and might explain the absence of *Botrytis* in greenhouse roots.

Interestingly, some beneficial microorganisms were enriched in S root samples such as *Rhizobiales* and *Glomerales*. The mycorrhizal intensity and the abundance of *Glomeromycota* sequences increased in roots of CS/RGM plants grown in S soils compared to AS ones. Arbuscular mycorrhizal fungi (AMF) are well studied root-associated fungi from the *Glomeromycota* division forming mutualistic symbiotic association with most of the terrestrial plants as well as grapevine (Trouvelot et al., 2015; Holland et al., 2016). Soil is an important factor driving the association between AMF and grapevine (Schreiner and Mihara, 2009). No affiliation was found in vineyard with *Glomeromycota* in S soil, while 0.02% of total fungal phyla in AS soil belonged to *Glomeromycota*. However, the choice of the primer is known to lead to a taxa bias, particularly for *Glomeromycota* strains (Bettenfeld et al., 2021) and we can hypothesize that our primers did not enable the identification of some species from this family. Nevertheless, AMF colonized the roots in each of the symptomatic conditions in greenhouse, suggesting either the presence of indigenous fungi from *Glomeromycota* division in the young vines obtained from nursery, or the proliferation of this undetected taxa during the greenhouse experiment. Before planting, root fungal microbiome of young CS/1103P and CS/RGM rootstocks were composed of *Ascomycota*, *Basidiomycota* and *Mortierellomycota* only, suggesting the likelihood of the second hypothesis.

Soil physicochemical properties are known to affect grapevine development (Conradie, 1986; Echenique et al., 2007). Furthermore, the influence of soil physicochemical parameters to modify the root associated microbial communities of grapevines was demonstrated (Karagöz et al., 2012; Zarraonaindia et al., 2015; Berlanas et al., 2019). Even if it is tough and sensitive to estimate the natural soil microbiome contribution to grapevine growth, here the novelty of this study was to compare two soils having similar physicochemical features with different microbial composition and functionality. During the greenhouse experiment, soil status had a significant effect on some growth parameters and chlorophyll content of both scion/rootstock combinations during the 2019 experiment only. This was probably due to higher temperatures in the greenhouse in 2019 compared to 2020, which may have produced conditions similar to those in the vineyard conditions (data not shown). This would suggest that the effect of the soil microbial composition on the growth of plants cannot be easily reproduced on young plants in greenhouse.

In terms of microbiome data, our results suggest that the rootstock genotype, in addition to soil composition, could be considered a driver of cultivable bacterial and fungal populations from the rhizosphere compartment. MALDI-TOF-MS revealed different bacterial diversity of cultivable population depending on the rootstock and soil status. Regarding sequencing results, no significant difference of bacterial diversity metrics in rhizosphere or roots was seen depending on the rootstock but fungal communities appeared to be dependent on it. Some orders were found to be enriched specifically in one scion/rootstock combination, such as *Cytophagales* in S-1103P. Moreover, the rootstocks showed differences in the abundance of fungal genera related to grapevine disease, which might be related to the metabolic composition of the roots. Modulation of the root-associated microbiome is known to be dependent on plant molecules, and some are believed to suppress some potential diseases (Pascale et al., 2020). The effect of grapevine rootstock on bacterial and/or fungal communities of the aboveground compartments has been previously investigated (D’Amico et al., 2018; Marasco et al., 2018; Berlanas et al., 2019; Swift et al., 2021; Vink et al., 2021; Marasco et al., 2022). Although some studies demonstrated that rootstock genotype shaped the microbial community structure (D’Amico et al., 2018; Marasco et al., 2022; Nerva et al., 2022), others were less definitive (Swift et al., 2021; Vink et al., 2021).

Moukarzel et al. (2021) showed that the rootstock drives the arbuscular mycorrhizal community structure. As described above, mycorrhization intensity increased when CS/RGM plants grew in S soils compared to AS soils, but the difference was not significant for CS/1103P. This result might be consistent with previous results showing that RGM cuttings exudated higher levels of strigolactones-like compounds than 1103P when subjected to nitrogen deficiency (Cochetel et al., 2018). Strigolactones have been shown to be involved in AMF symbiosis (Yoneyama and Brewer, 2021). The ability of plant roots to attract and select microbes from the soil is ruled by the different signaling compounds, primary (e.g., carbohydrates, organic acids, and amino acids), and secondary (e.g., glucosinolates, and flavonoids) metabolites exudated by the rootstock towards soil (Sasse et al., 2018; Vives-Peris et al., 2020). Marastoni et al. (2019) unveiled the different root exudate compositions of distinct grapevine rootstocks. Root exudates have also been investigated in copper toxicity (Marastoni et al., 2019) and iron deficiency (Marastoni et al., 2020) conditions but no studies have investigated the impact of grapevine exudates on microbial communities. Potential metabolic diversity, based on potential root exudate consumption, such as amines, amino-acids, carbohydrates, carboxylic acids, phenolic compounds, and polymers, revealed distinct profiles with higher activities in S soils compared to AS soils for both rootstock combinations. These findings suggested higher effect from the soil status than rootstock genotype on the microbial functional diversity, while no significant effect by both rootstock or soil factors was observed on the taxonomic diversity or richness in the rhizosphere compartment for bacterial community. In fact, the soil status was more important than rootstock genotype in driving the fungal diversity, besides year of experiment and compartment effects. Since all the previous studies showing the impact of the rootstock on the root microbiota were conducted on older plants in the vineyard, we can hypothesize that the impact of the rootstock is not seen on very young plants.

## Conclusion

This study provides knowledge on the complex interaction between soil microbiome and grapevine roots of young grapevine plants. It confirms the importance of the soil composition on the microbiome associated with belowground compartments, while rootstock of these young plants showed minor impact on microbial communities. Even if the decline observed in the vineyard could not be reproduced in the greenhouse, soils used for the controlled experiment altered the root and the rhizosphere microbiomes for the two scion/rootstock plants. Fungal communities were therefore more impacted by the soil status than the bacteria, suggesting their determinant role in the future growth of these young grapevines. This work also highlights the difficulty in defining a so-called soil microbial “dysbiosis” or, on the contrary, a “good” soil microbiome, capable of promoting the growth of healthy plants. It appeared that the community structure was different and that asymptomatic soils contained less putative pathogenic fungi than symptomatic soils. But the main question still remains: is this dysbiosis the cause or the consequence of the observed decline? To answer this question, the longer-term effects on the plants will need to be monitored. Interestingly, when plants are subjected to symptomatic soils with more fungal pathogens, they increase their interaction with beneficial microbes, following the cry-for-help concept (Rolli et al., 2021). It might therefore be interesting to search for plant growth promoting bacteria (PGPB) and biocontrol agents in declining soils rather than in healthy soils. This should be taken into account in the development and use of targeted agriculture applications, especially in light of grapevine decline.

## Data availability statement

The data presented in the study are deposited in the NCBI Bioproject repository, accession number PRJNA798301. The data have been publicly released. https://www.ncbi.nlm.nih.gov/bioproject/PRJNA798301.

## Author contributions

RD, VL, IM-P, and NO conceived the study. RD managed the greenhouse experiment and the sampling, he performed DNA extraction and microbiological analyses, and prepare the figures and tables. RD, GM, PB, PV, EM, NO, and VL contributed to the data collection and analysis. BM performed the sequencing and contributed to bioinformatics analyses. RD, LA, BM, and SC did the bioinformatics analyses. RD and SC performed the DOPE-FISH experiments. RD, PB, and IM-P contributed to MALDI-TOF-MS experiments and data analyses. RD and VL wrote the manuscript. All authors critically reviewed and edited the manuscript.

## Funding

This work was supported by FranceAgrimer/CNIV funded as part of the program ‘Plan National Dépérissement du Vignoble’ within the project Vitirhizobiome (grant number 2018–52537).

## Conflict of interest

The authors declare that the research was conducted in the absence of any commercial or financial relationships that could be construed as a potential conflict of interest.

## Publisher’s note

All claims expressed in this article are solely those of the authors and do not necessarily represent those of their affiliated organizations, or those of the publisher, the editors and the reviewers. Any product that may be evaluated in this article, or claim that may be made by its manufacturer, is not guaranteed or endorsed by the publisher.

## Footnotes

^1^ www2.dijon.inra.fr/mychintec/Mycocalc-prg/download.html
