# Supplementary material for: Soil composition and rootstock genotype drive the root associated microbial communities in young grapevines
Source: Front Microbiol. 2022 Nov 10;13:1031064. doi: 10.3389/fmicb.2022.1031064 (PMC9685171; doi:10.3389/fmicb.2022.1031064)
Supplement: Supplementary file 1 [file Presentation_1.pdf]

Supplementary Material

1 Supplementary Tables

Supplementary Table 1. Primers used for 16S rRNA gene, and nested-based PCR for ITS amplifications. Specific overhang Illumina adapters are underlined.

Primer	Primer sequence (5’ → 3’)
799f	TCGTCGGCAGCGTCAGATGTGTATAAGAGACAGAACMGGATTAGATACCCKG
1175r	GTCTCGTGGGCTCGGAGATGTGTATAAGAGACAGACGTCRTCCCCDCCTTCCTC
ITS1F	CTTGGTCATTTAGAGGAAGTAA
TW13	GGTCCGTGTTTCAAGACG
ITS31_NeXTf	TCGTCGGCAGCGTCAGATGTGTATAAGAGACAGCATCGATGAAGAACGCAG
ITS32_NeXTf	TCGTCGGCAGCGTCAGATGTGTATAAGAGACAGCAACGATGAAGAACGCAG
ITS33_NeXTf	TCGTCGGCAGCGTCAGATGTGTATAAGAGACAGCACCGATGAAGAACGCAG
ITS34_NeXTf	TCGTCGGCAGCGTCAGATGTGTATAAGAGACAGCATCGATGAAGAACGTAG
ITS35_NeXTf	TCGTCGGCAGCGTCAGATGTGTATAAGAGACAGCATCGATGAAGAACGTGG
ITS4_NeXTr	GTCTCGTGGGCTCGGAGATGTGTATAAGAGACAGTCCTCCGCTTATTGATATGC
ITS43S_NeXTr	GTCTCGTGGGCTCGGAGATGTGTATAAGAGACAGTCCTSSSCTTATTGATATGC'
5.8S-Fun_NeXTf	TCGTCGGCAGCGTCAGATGTGTATAAGAGACAGGGAACTTTYRRCAAYGGATCWCT
ITS4-Fun_NeXTr	GTCTCGTGGGCTCGGAGATGTGTATAAGAGACAGAAAGCCTCCGCTTATTGATATGCTTAART
ITS43S-Fun_NeXTr	GTCTCGTGGGCTCGGAGATGTGTATAAGAGACAGAAAGCCTSSSCTTATTGATATGCTTAART

Supplementary Table 2. Conditions cycling for amplicon preparation of 16S and ITS libraries.

Primer pairs	Initial denaturation	Number of cycles	Denaturation	Annealing	Extension	Final extension
799f and 1175r	5 min at 95°C	32	30 s at 95°C	30 s at 55°C	45 s at 72°C	5 min at 72°C
ITS1F and TW13	5 min at 95°C	30	30 s at 95°C	30 s at 50°C	90 s at 72°C	5 min at 72°C
ITS3_Mix and ITS4_Mix	3 min at 95°C	30	30 s at 95°C	30 s at 60°C	45 s at 72°C	5 min at 72°C
5.8S-Fun_NeXTf and ITS5_Mix	3 min at 95°C	42	35 s at 95°C	30 s at 53°C	42 s at 72°C	5 min at 72°C

Supplementary Table 3. Probes used for DOPE-FISH, with the associated modifications and target.

Oligoname	Sequence (5’ -> 3’)	Modifications	Target
**EUB338**	GCTGCCTCCCGTAGGAGT	5’-CY3<br>3’-CY3	All bacteria
**EUB338II**	GCAGCCACCCGTAGGTGT	5’-CY3<br>3’-CY3	
**EUB338III**	GCTGCCACCCGTAGGTGT	5’-CY3<br>3’-CY3	
**NONEUB**	ACTCCTACGGGAGGCAGC	5’-CY5<br>3’-CY5	Non-negative control
**NONEUB**	ACTCCTACGGGAGGCAGC	5’-CY3<br>3’-CY3	
**Chit**	GGAAGGTTGCGTACGTGT	5’-CY5<br>3’-CY5	*Chitinophaga* (16S)
**Rhizo4**	GCGTTATTCCGTAGTCAAGG	5’-CY5<br>3’-CY5	*Rhizobium* (16S)
**Pseu4**	CACCAGGTACAAGTACCCGT	5’-CY5<br>3’-CY5	*Pseudomonas* (16S)
**Pce**	CCCATCGCATCTAACAAT	5’-CY5<br>3’-CY5	*Burkholderia* (23S)

Supplementary Table 4. Diversity metrics of bacterial and fungal communities for each condition, regardless of sampling year. Data are presented as means ± SE, n = 6 biological replicates. Letters indicate significant differences between the conditions (i.e. compartment x rootstock genotype x soil status) using pairwise comparison, $p<0.05$.

		Rootstock	Soil	Richness (Observed ASVs)	α-diversity (Simpson's index)
Bacteria	Bulk	1103P	AS	783 ± 55 a	0.996 ± 0.001 a
			S	709 ± 61 a	0.995 ± 0.001 a
		RGM	AS	681 ± 23 a	0.995 ± 0.001 a
			S	774 ± 21 a	0.994 ± 0.002 a
	Rhizosphere	1103P	AS	672 ± 37 a	0.996 ± 0.001 a
			S	792 ± 50 a	0.997 ± 0.001 a
		RGM	AS	691 ± 49 a	0.996 ± 0.001 a
			S	772 ± 15 a	0.997 ± 0.001 a
	Roots	1103P	AS	400 ± 34 bc	0.955 ± 0.013 cd
			S	360 ± 55 bc	0.971 ± 0.005 be
		RGM	AS	329 ± 19 bc	0.958 ± 0.008 ce
			S	296 ± 24 c	0.942 ± 0.013 d
	Vineyard		AS	788 ± 113 a	0.995 ± 0.001 a
			S	666 ± 45 a	0.996 ± 0.001 a
	Nursery	1103P		427 ± 14 bc	0.981 ± 0.001 ab
		RGM		493 ± 40 c	0.979 ± 0.004 ab
Fungi	Bulk	1103P	AS	476 ± 13 a	0.980 ± 0.003 ab
			S	465 ± 22 a	0.979 ± 0.003 ab
		RGM	AS	425 ± 20 a	0.969 ± 0.009 ab
			S	469 ± 13 a	0.977 ± 0.004 ab
	Rhizosphere	1103P	AS	477 ± 16 a	0.978 ± 0.007 ab
			S	468 ± 24 a	0.979 ± 0.004 ab
		RGM	AS	454 ± 24 a	0.978 ± 0.005 ab
			S	489 ± 12 a	0.980 ± 0.003 ab
	Roots	1103P	AS	205 ± 41 b	0.954 ± 0.008 f
			S	218 ± 47 b	0.969 ± 0.005 ac
		RGM	AS	171 ± 29 b	0.937 ± 0.005 e
			S	203 ± 33 b	0.961 ± 0.005 cf
	Vineyard		AS	453 ± 21 a	0.983 ± 0.002 ab
			S	484 ± 23 a	0.987 ± 0.001 b
	Nursery	1103P	1103P	132 ± 6 b	0.923 ± 0.006 de
		RGM	RGM	141 ± 11 b	0.911 ± 0.007 d

Supplementary Table 5. Eco-Plates measurements (Area Under Curve (AUC), Simpson index (E) and richness (R) functionality at 96 hours post-incubation) for the rhizosphere samples in 2019 and 2020 experiments. The two rootstocks RGM and 1103P have been grown with S or AS soils. Data are presented as means ± SE, n = 3 biological replicates. Letters indicate significant differences between the conditions (i.e. rootstock genotype x soil status) using pairwise comparison based on student t or Wilcoxon tests, $p<0.05$.

	2019				2020			
	RGM		1103P		RGM		1103P	
	S	AS	S	AS	S	AS	S	AS
AUC	10.6 ± 0.4 b	9.5 ± 0.2 a	12 ± 0.5 c	9.6 ± 0.4 a	3.1 ± 0.3 B	3.8 ± 0.1 C	2.9 ± 0.1 B	2.6 ± 0.1 A
E	0.974 ± 0.005 b	0.967 ± 0.006 a	0.975 ± 0.006 b	0.978 ± 0.003 b	0.982 ± 0.019 B	0.877 ± 0.015 A	0.893 ± 0.032 A	0.866 ± 0.013 A
R	25 ± 1 c	19 ± 2.6 a	22.3 ± 3.2 ac	19.3 ± 3.1 a	9.7 ± 0.5 B	7.7 ± 0.2 A	7 ± 1.7 A	6.7 ± 0.6 A

Supplementary Table 6. Stem length, diameter, and chlorophyll content in leaves of grafted plants grown on S or AS soils at the end of each experiment (2019 and 2020). Data are presented as means ± SE, n = 10 individual plants (biological replicates). Letters indicate significant differences between the conditions (*i.e.* rootstock genotype x soil status) using pairwise comparison based on student t or Wilcoxon tests, $p<0.05$.

		Roots dry weight (g)	Shoot dry weight (g)	Total stem length (cm)	Stem diameter (cm)	Chlorophyll leaves content
2019	AS-1103P	8.2 ± 1.6 **a**	7.6 ± 0.5 **b**	68.9 ± 7.5 **c**	0.9 ± 0.1 a	28.1 ± 1.2 **c**
	S-1103P	6.3 ± 1.7 **ab**	6.1 ± 1.5 **a**	55.2 ± 13.8 **ab**	0.9 ± 0.2 a	21.4 ± 2 **a**
	AS-RGM	7.8 ± 1.2 **b**	7.6 ± 1.1 **b**	61.7 ± 8.6 **bc**	0.9 ± 0.2 a	27.9 ± 1.9 **c**
	S-RGM	7.4 ± 1.3 **ab**	7.0 ± 1.6 **ab**	50.3 ± 8 **a**	0.8 ± 0.2 a	25.8 ± 2 **b**
2020	AS-1103P	11.7 ± 2.6 a	9.6 ± 3.5 a	78.6 ± 14.9 a	0.9 ± 0.2 **ab**	20.9 ± 3.1 a
	S-1103P	12.5 ± 1.5 a	12.2 ± 3.9 a	78.5 ± 17.1 a	1 ± 0.1 **a**	21.9 ± 2.5 a
	AS-RGM	12.6 ± 1.3 a	11.2 ± 1.7 a	71.3 ± 13.9 a	0.8 ± 0.1 **b**	22 ± 3.7 a
	S-RGM	12.5 ± 2.2 a	10.4 ± 1.6 a	69.5 ± 13 a	0.8 ± 0.1 **b**	22 ± 3 a

Supplementary Table 7. List of the fungal genera, associated to grapevine diseases, searched through the samples.

	Associated disease	References*
Anthostoma	Eutypa	(Gramaje et al., 2018; Del Frari et al., 2019)
Botrytis	Grey mould	(González-Fernández et al., 2020)
Botryosphaeria	Botryosphaeria dieback	(Mondello et al., 2015)
Cadophora	Petri disease	(Gramaje et al., 2021)
Campylocarpon	Black foot	(Gramaje et al., 2018)
Cryptosphaeria	Canker, Eutypa	(Trouillas et al., 2010)
Cryptovalsa	Canker, Eutypa	(Niem et al., 2020; Trouillas et al., 2010)
Cytospora	Canker, Eutypa	(Lawrence et al., 2017)
Curvularia	Canker	(Bahmani et al., 2021)
Cylindrocladiella	Black foot	(Gramaje et al., 2018)
Dactylonectria	Black foot	(Gramaje et al., 2018)
Diaporthe	Phomopsis dieback	(Yan et al., 2013)
Diatrype	Eutypa dieback	(Trouillas et al., 2010)
Diatrypella	Eutypa dieback	(Gramaje et al., 2018)
Diplodia	Botryosphaeria dieback	(Gramaje et al., 2018)
Dothiorella	Botryosphaeria dieback	(Gramaje et al., 2018)
Eutypa	Eutypa dieback	(Cardot et al., 2019)
Eutypella	Eutypa dieback	(Gramaje et al., 2018)
Fomitiporia	Esca	(Gramaje et al., 2018)
Fomitiporella	Esca	(Gramaje et al., 2018)
Fusicoccum	Phomopsis dieback	(Gramaje et al., 2018)
Ilyonectria	Black foot	(Lade et al., 2022)
Inocutis	Esca	(Gramaje et al., 2018)
Inonotus	Esca	(Gramaje et al., 2018)

Lasiodiplodia	Botryosphaeria dieback	(Gramaje et al., 2018)
Neofusicoccum	Botryosphaeria dieback	(Gramaje et al., 2018)
Neonectria	Black foot	(Gramaje et al., 2018)
Neoscytalidium	Botryosphaeria dieback	(Gramaje et al., 2018)
Phaeobotryosphaeria	Botryosphaeria dieback	(Gramaje et al., 2018)
Phaeoacremonium	Petri disease	(Lade et al., 2022)
Phaeomoniella	Petri disease	(Lade et al., 2022)
Phellinus	Esca	(Gramaje et al., 2018)
Pleurostoma	Petri disease	(Gramaje et al., 2018)
Spencermartinsia	Botryosphaeria dieback	(Gramaje et al., 2018)
Stereum	Esca	(Gramaje et al., 2018)
Thelonectria	Black foot	(Gramaje et al., 2018)

*References

Bahmani, Z., Abdollahzadeh, J., Amini, J., and Evidente, A. (2021). Biscogniauxia rosacearum the charcoal canker agent as a pathogen associated with grapevine trunk diseases in Zagros region of Iran. *Sci. Rep.* 11, 14098. https://doi.org/10.1038/s41598-021-93630-w

Cardot, C., Mappa, G., La Camera, S., Gaillard, C., Vriet, C., Lecomte, P., et al. (2019). Comparison of the molecular responses of tolerant, susceptible and highly susceptible grapevine cultivars during interaction with the pathogenic fungus Eutypa lata. *Front. Plant Sci.* 10, 1–18. https://doi.org/10.3389/fpls.2019.00991

González-Fernández, E., Piña-Rey, A., Fernández-González, M., Aira, M.J., and Rodríguez-Rajo, F.J. (2020). Identification and evaluation of the main risk periods of Botrytis cinerea infection on grapevine based on phenology, weather conditions and airborne conidia. *J. Agric. Sci.* 158, 88–98. https://doi.org/10.1017/S0021859620000362

Gramaje, D., Eichmeier, A., Spetik, M., Carbone, M. J., Bujanda, R., Vallance, J., and Rey, P. (2022). Exploring the temporal dynamics of the fungal microbiome in rootstocks, the lesser-known half of the grapevine crop. *J. Fungi* 8(5), 421. https://doi.org/10.3390/jof8050421

Gramaje, D., Urbez-Torres, J. R., and Sosnowski, M. R. (2018). Managing grapevine trunk diseases with respect to etiology and epidemiology: current strategies and future prospects. *Plant Dis*., 102(1), 12-39. https://doi.org/10.1094/PDIS-04-17-0512-FE

Lade, S.B., Štraus, D., and Oliva, J. (2022). Variation in fungal community in grapevine (Vitis vinifera) nursery stock depends on nursery, variety and rootstock. *J. Fungi* 8, 47. https://doi.org/10.3390/jof8010047

Lawrence, D.P., Travadon, R., Pouzoulet, J., Rolshausen, P.E., Wilcox, W.F., and Baumgartner, K., (2017). Characterization of Cytospora isolates from wood cankers of declining grapevine in North America, with the descriptions of two new Cytospora species. *Plant Pathol.* 66, 713–725. https://doi.org/10.1111/ppa.12621

Mondello, V., Spagnolo, A., Larigno, P., Clément, C., and Florence, F. (2015). Phytoprotection potential of Fusarium proliferatum for control of Botryosphaeria dieback pathogens in grapevine. *Phytopathol. Mediterr*.58, 293–306.

Niem, J.M., Billones-Baaijens, R., Stodart, B., and Savocchia, S. (2020). Diversity profiling of grapevine microbial endosphere and antagonistic potential of endophytic Pseudomonas against grapevine trunk diseases. *Front. Microbiol*.. 11, 1–19. https://doi.org/10.3389/fmicb.2020.00477

Del Frari, G., Gobbi, A., Aggerbeck, M. R., Oliveira, H., Hansen, L. H., and Ferreira, R. B. (2019). Characterization of the wood mycobiome of Vitis vinifera in a vineyard affected by esca. Spatial

distribution of fungal communities and their putative relation with leaf symptoms. *Front. Plant Sci.* 10, 910. https://doi.org/10.3389/fpls.2019.00910

Trouillas, F.P., Úrbez-Torres, J.R., and Gubler, W.D. (2010). Diversity of diatrypaceous fungi associated with grapevine canker diseases in California. *Mycologia* 102, 319–336. https://doi.org/10.3852/08-185

Yan, J.Y., Xie, Y., Zhang, W., Wang, Y., Liu, J.K., Hyde, K.D., et al. (2013). Species of Botryosphaeriaceae involved in grapevine dieback in China. *Fungal Divers.* 61, 221–236. https://doi.org/10.1007/s13225-013-0251-8

Supplementary Table 8. Estimation of the root colonization by arbuscular mycorrhizal fungi using microscopy-staining method, and ITS-sequencing at the end of the 2020's experiment in greenhouse. Frequency of mycorrhization (F%) and intensity of mycorrhization (M%) were estimated on fine roots using Trouvelot *et al.* (1986) method. Relative abundance of *Glomeromycota* (%) was obtained after sequencing of root samples for RGM and 1103P rootstocks grown in asymptomatic (AS) and symptomatic (S) soils. Data are presented as means ± SE, with n = 10 individual plants (biological replicates) for F and M indexes and n=3 for sequencing data. Letters indicate significant differences between the conditions (i.e. rootstock genotype x soil status) using pairwise comparison, $p<0.05$.

	RGM		1103P	
	S	AS	S	AS
F (%)	96.7 ± 4.7 a	96 ± 7.2 a	88 ± 12.2 a	87.3 ± 10.9 a
M (%)	52.5 ± 13.2 b	34.8 ± 12.5 a	39.7 ± 10.7 ab	25.5 ± 5.7 a
Relative abundance *Glomeromycota* (%)	16.69 ± 4.79 b	2.35 ± 0.45 a	11.26 ± 3.70 a	7.09 ± 2.38 a

2 Supplementary Figures

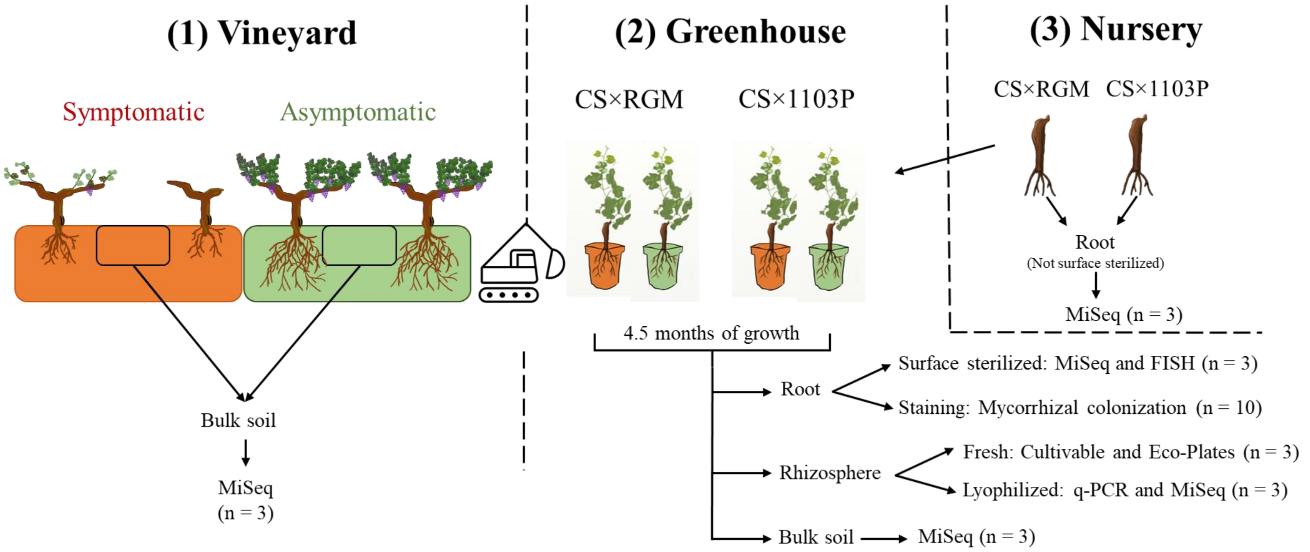

Supplementary Figure 1. Schematic overview of the experimental design carried out, from the vineyard soil excavation to the greenhouse setup. (**1**) Soils from vineyard were sampled in April at the beginning of the greenhouse experiment (n = 3 × 2 soils). (**2**) Grafted plants were planted in the pots with the two soils and after 4.5 months of growth, they were phenotyped (n = 10), and samples of roots, rhizosphere and bulk soils were harvested as described in the methods (n = 3 × 4 treatments). (**3**) Roots from bare-root plants from the nursery were sampled before planting in April (n = 3 × 2 genotypes). This experiment has been repeated twice, in 2019 and 2020 with new soil from vineyard and new nursery plants.

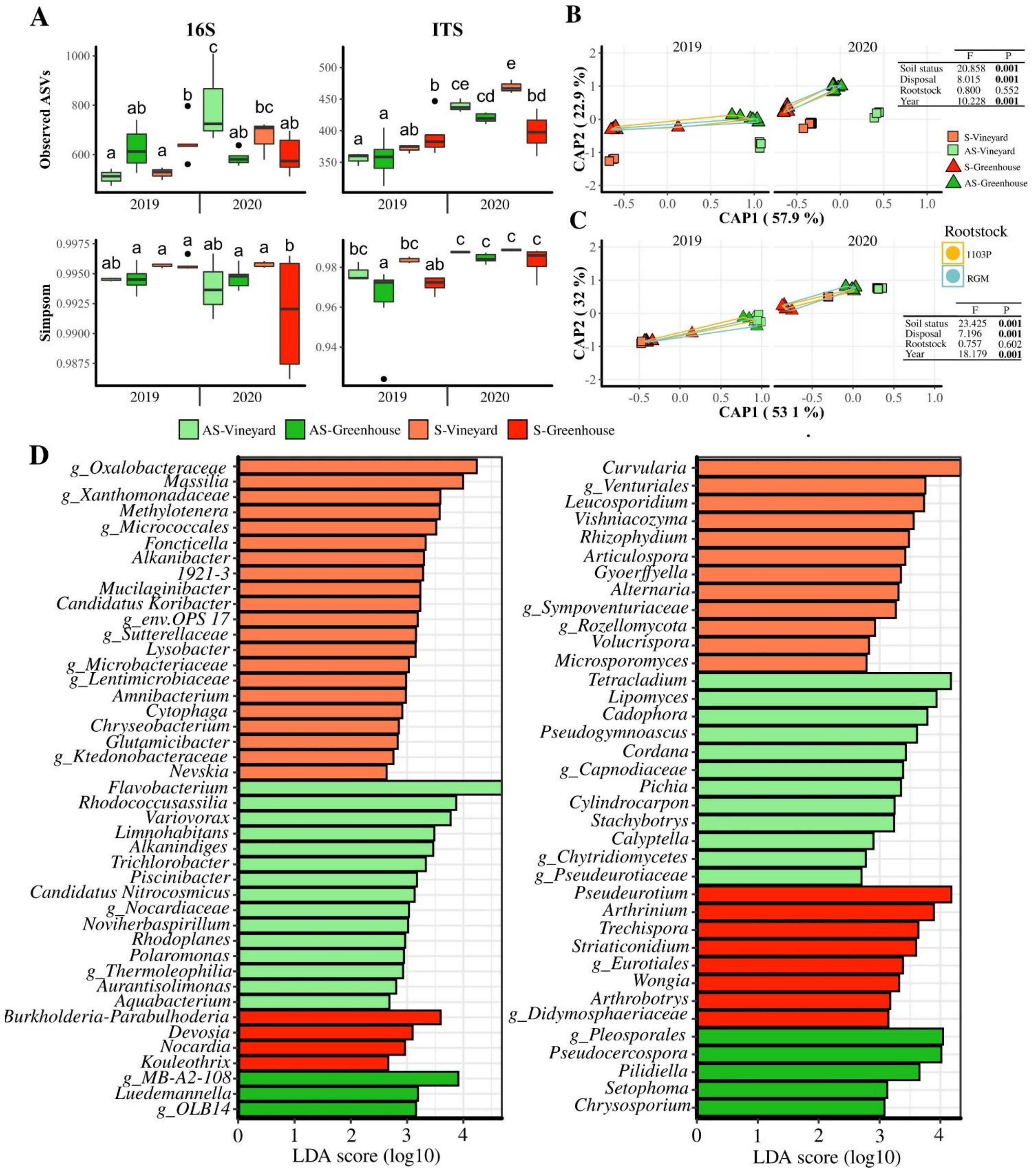

Supplementary Figure 2. (**A**) Diversity metrics (i.e., observed ASVs and Simpson's diversity index) of bulk symptomatic and asymptomatic soils from different disposals (vineyard and greenhouse) across the two sampling years for bacterial and fungal communities. Different letters indicate significantly different means according to pairwise comparisons based on Wilcoxon or Student t tests ($p < 0.05$). CAP of bulk symptomatic and asymptomatic soils from vineyard and greenhouse, related to 1103P and RGM rootstocks, repeated twice (2019 and 2020) for (**B**) bacterial and (**C**) fungal communities. Tables represent PERMANOVA by terms from "capscale" function (permutations = 9,999). (**D**) LEfSe for

bacterial and fungal genera on bulk and vineyard samples regardless of sampling year and rootstock genotype.

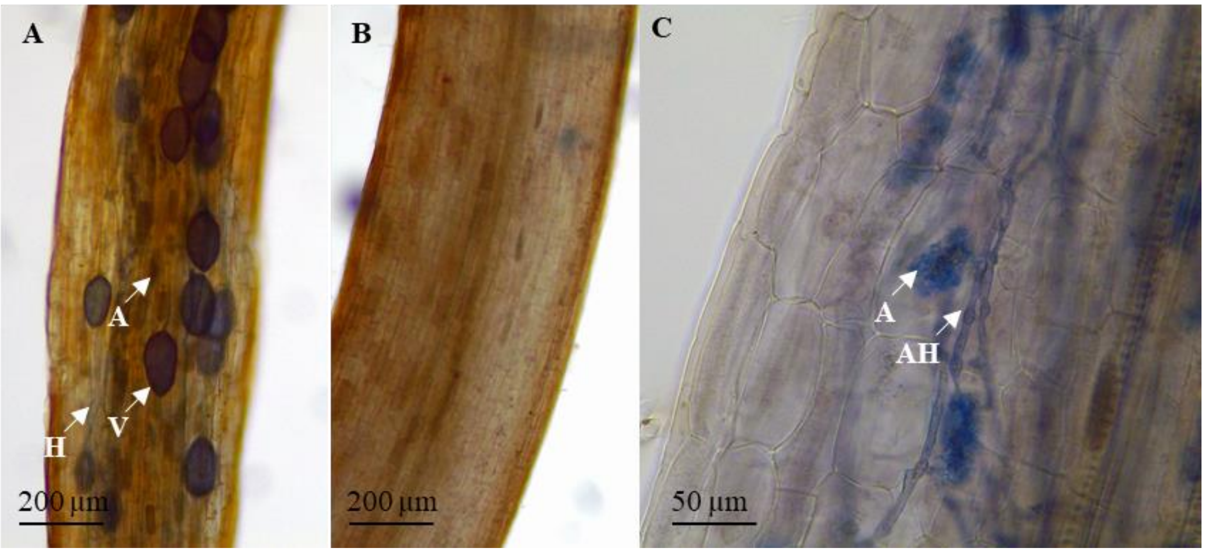

Supplementary Figure 3. Photomicrographs of grapevine fine roots stained with black ink. (**A**) Mycorrhized root where hyphae, arbuscules and vesicles are visible, (**B**) Root with absence of mycorrhization, and (C) visualization of hyphae and arbuscules at a higher magnitude. H = Hyphae, V = Vesicle, A = Arbuscule, AH = Aseptate hyphae.

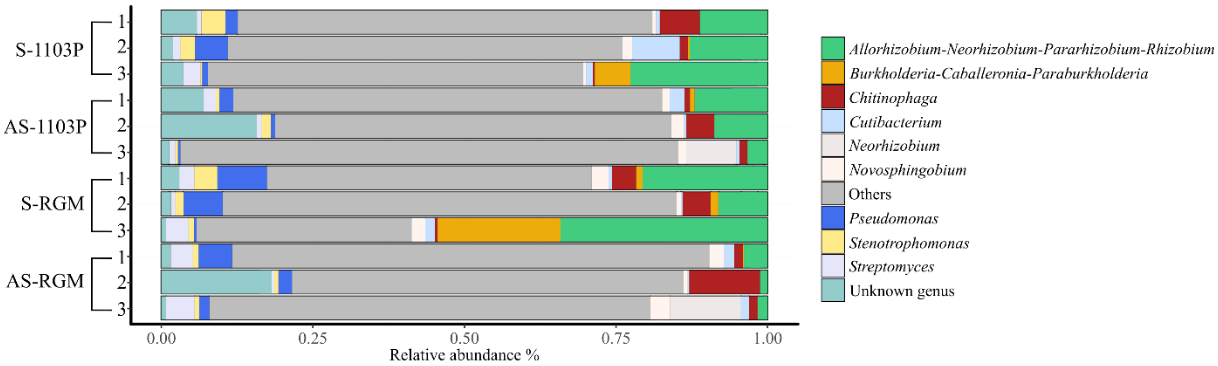

Supplementary Figure 4. Relative abundance of the 10 most represented bacterial genera identified in roots after sequencing in 2019 experiment. The three biological replicates (individual plants) are presented for each condition: plants grafted on 1103P or RGM rootstock and grown on S or AS soils. The less represented genera were grouped in “Others”.

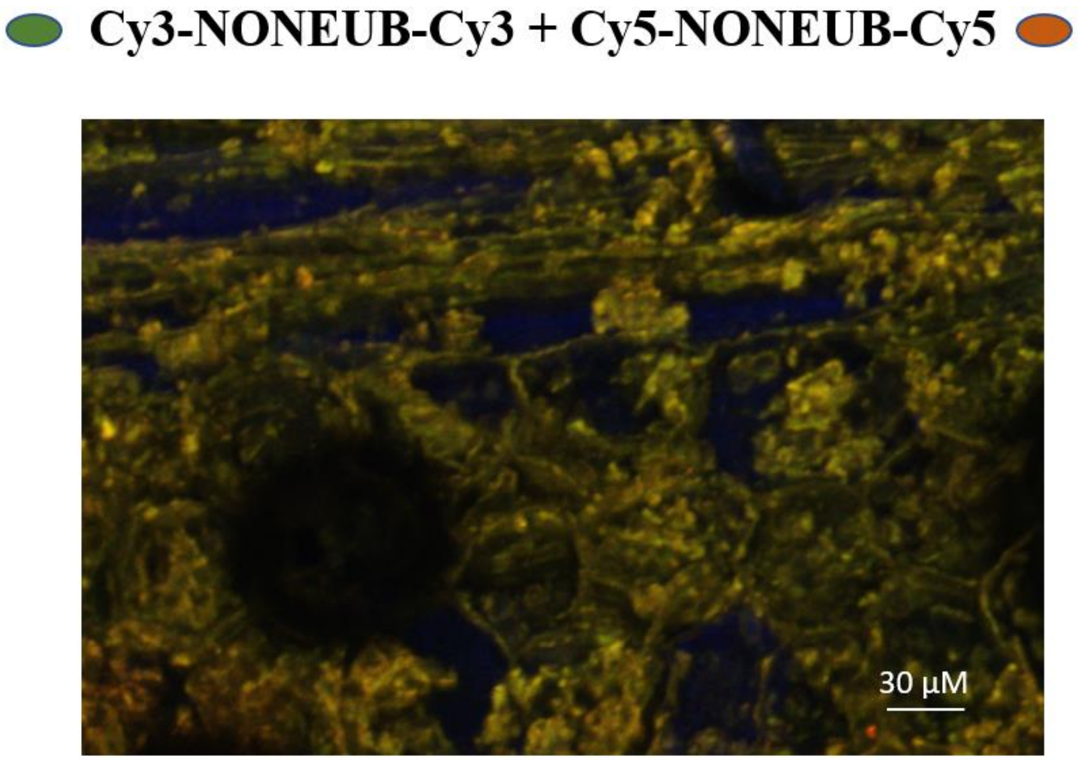

Supplementary Figure 5. Visualization of the root endosphere using negative NONEUB probe.
